# QM/Classical Modeling of Surface Enhanced Raman Scattering Based on Atomistic Electromagnetic Models

**DOI:** 10.1021/acs.jctc.3c00177

**Published:** 2023-06-06

**Authors:** Piero Lafiosca, Luca Nicoli, Luca Bonatti, Tommaso Giovannini, Stefano Corni, Chiara Cappelli

**Affiliations:** † Scuola Normale Superiore, Piazza dei Cavalieri 7, 56126 Pisa, Italy; ‡ Dipartimento di Scienze Chimiche, Università di Padova, via Marzolo 1, 35131 Padova, Italy; ¶ Istituto di Nanoscienze del Consiglio Nazionale delle Ricerche CNR-NANO, via Campi 213/A, 41125 Modena, Italy; § LENS (European Laboratory for Non-Linear Spectroscopy), via N. Carrara 1, 50019 Sesto Fiorentino, Italy

## Abstract

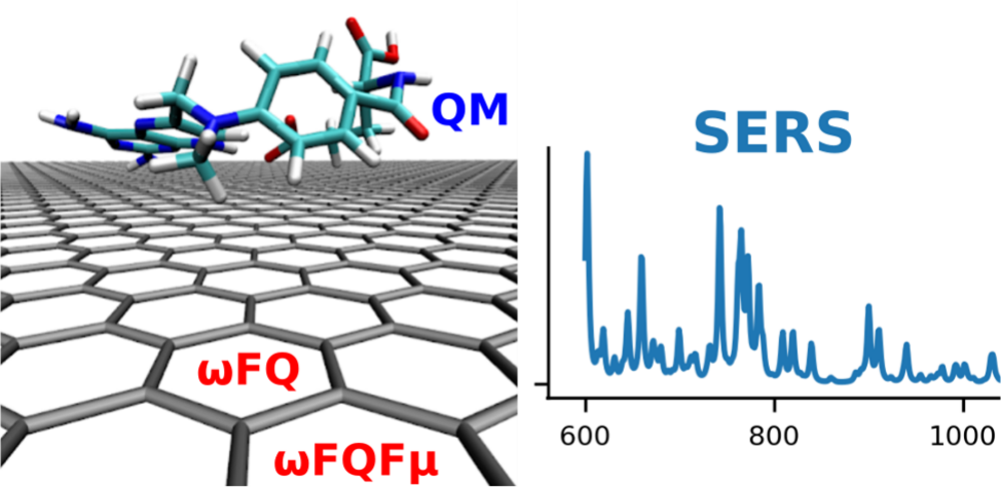

We present quantum mechanics (QM)/frequency dependent fluctuating charge (QM/ωFQ) and fluctuating dipoles (QM/ωFQFμ) multiscale approaches to model surface-enhanced Raman scattering spectra of molecular systems adsorbed on plasmonic nanostructures. The methods are based on a QM/classical partitioning of the system, where the plasmonic substrate is treated by means of the atomistic electromagnetic models ωFQ and ωFQFμ, which are able to describe in a unique fashion and at the same level of accuracy the plasmonic properties of noble metal nanostructures and graphene-based materials. Such methods are based on classical physics, i.e. Drude conduction theory, classical electrodynamics, and atomistic polarizability to account for interband transitions, by also including an ad-hoc phenomenological correction to describe quantum tunneling. QM/ωFQ and QM/ωFQFμ are thus applied to selected test cases, for which computed results are compared with available experiments, showing the robustness and reliability of both approaches.

## 1 Introduction

Surface-enhanced Raman scattering (SERS) takes advantage of the giant enhancement of the Raman scattering cross section of a target molecule in the proximity of plasmonic nanostructured materials.^1^ Enhancement factors (i.e., the ratio between the Raman intensity for the nanoaggregate and the isolated/solvated target molecule) can reach values up to 10^10–11^,^2−4^ thus allowing single molecule detection, down to a submolecular resolution.^5^ For these reasons, SERS has gained popularity and is widely used in a plethora of applications^6−11^ because it inherits the general advantages of classical Raman spectroscopy, solves its main weakness, consisting of generally low scattering amplitudes, and add, possibly, spatial resolution on the nanometer scale and below.^12−14^ From the physicochemical point of view, it is generally accepted^3,15−17^ that SERS enhancement results from the combination of two factors: the so-called electromagnetic (EM) effect (yielding enhancements up to 10^7–8^), which is caused by the excitation of surface plasmons in the substrate, which leads to a strong induced electric field in its proximity, and the so-called chemical (CT) enhancement, for which a holistic theoretical explanation is still missing. CT is associated with 10^2–3^ enhancement factors and is mainly ascribed to charge-transfer excitations between the analyte and the substrate.

Metal nanostructures, such as metal nanoparticles (NPs), have been the most used substrates for SERS because they provide highly confined plasmons and huge enhanced electric field on their surfaces,^15,18−24^ also thanks to a substantial advancement in experimental techniques for manipulating the nanoscale.^25−29^ Indeed, specific morphological arrangements can be designed, giving rise to hot-spots, i.e. regions in space where the electric field is extremely confined and enhanced.^5,12,13,30^ Recently, there has been increasing interest in designing novel substrates characterized by high chemical inertness to be used in the investigation of biochemical species. In this context, recent developments of SERS substrates based on carbon allotropes such as graphene and carbon nanotubes (CNTs) are worth being mentioned.^31−33^

A theoretical understanding of the SERS mechanisms can be particularly useful not only for the interpretation of experimental spectra but also for the in-silico design of novel materials and morphologies that can maximize spectral enhancement factors for a specific analyte. For this reason, in the past years, various methodologies have been proposed to simulate SERS signals.^34−46^ The huge dimension of typical SERS substrates (tens/hundreds of nanometers) makes a full quantum mechanical (QM) description of the molecule–substrate system unfeasible, although small-size model systems can be exploited to deal with specific features of the SERS phenomenon (mainly related to the CT mechanism).^47−50^ To solve this problem, multiscale approaches can be used, where the analyte is described at the QM level, while the substrate is treated classically.^34,35,37−41,51−59^ In particular, the nanostructured material can be modeled as a continuum medium, defined in terms of its complex-valued permittivity, or by retaining its atomistic nature. Remarkably, in the latter case a precise description of complex geometries, even characterized by geometrical defects, is obtained.^60,61^ Note that both approaches neglect CT effects; however, for usual SERS substrates, the EM enhancement is several orders of magnitude higher than the CT.^62^

In this paper, we present QM/ωFQ and QM/ωFQFμ fully atomistic multiscale QM/classical approaches to simulate SERS spectra, where the analyte is treated quantum-mechanically. To describe the nanostructured materials, we exploit a family of atomistic models that we have recently developed, which are able to correctly reproduce experimental and ab initio plasmonic features of metal nanoparticles (ωFQ and ωFQFμ),^61,63^ and graphene-based nanostructures (ωFQ).^64^ These approaches are based on classical physics and text-book concepts, such as Drude conduction theory, classical electrodynamics, and atomistic polarizability, to account for interband transitions. In addition, ad-hoc phenomenological correction to describe quantum tunneling is included in the model, to deal with nanojunctions and aggregates in which hot-spots may originate. Within ωFQ and ωFQFμ, each atom of the nanostructure is endowed with a frequency-dependent complex-valued charge and supplemented by a complex-valued dipole in ωFQFμ, which responds to the external radiation, thus mimicking the oscillating electron density.

Different from previous classical atomistic approaches,^34−36,42−46,59^ Drude conduction, i.e. intraband transitions, is taken into account by means of the equation of motion of the charges placed on each atom, while induced dipoles are included so as to model interband transitions.^61^ This permits us to correctly catch the physics underlying the plasmonics of generic *s*, *p*, and *d* metallic systems by also allowing for a physical dissection of the two contributions. Moreover, ωFQ(Fμ) is able to physically account for the quantum tunneling between nearby nanoparticles by exponentially modulating Drude conductance as a function of atom–atom distances, which is particularly relevant for nanoaggregates and nanojunctions, where plasmonic hot-spots are created.^61,63^ Finally, it is worth noting that ωFQ is the only atomistic classical approach to date able to physically describe graphene plasmonics in terms of its fundamental physical parameters, such as the Fermi energy, relaxation time, and two-dimensional electron density.^60,64−66^

Here, ωFQ and ωFQFμ are coupled to a density functional theory (DFT) treatment of the QM portion and the resulting QM/ωFQ and QM/ωFQFμ methods are further extended to the calculation of complex molecular polarizabilities through the linear response theory. The robustness of the approaches is showcased through their application to pyridine adsorbed on noble metal NPs. Thanks to the generality of both approaches, we also apply them to the simulation of graphene-enhanced Raman spectroscopy (GERS) by exploiting graphene-based nanostructures as enhancing substrates. For large pristine graphene sheets, the absence of sharp edges is usually connected to the low enhancement factors reported for GERS.^67,68^ Nevertheless, suitably engineered graphene-based nanostructures may enhance by several orders of magnitude both the induced electric field and its spatial confinement.^60^

The paper is organized as follows. The first section recalls the theoretical foundations of ωFQ and ωFQFμ and presents their coupling with a DFT Hamiltonian for the ground state and linear response theory. We note that the EM field associated with the induced density in the nanoparticle is neither a purely real nor a purely imaginary perturbation. Therefore, we exploit a general complex linear response formulation. QM/ωFQFμ is then applied to simulate SERS spectra of pyridine adsorbed on different plasmonic substrates (silver, gold, and graphene). Finally, GERS of a widely used anticancer drug, i.e. methotrexate, adsorbed on a graphene disk, is taken as a case study to showcase the potentialities of the method. Summary, conclusions, and future perspectives end the paper.

## 2 Theory

### 2.1 ωFQ and ωFQFμ

ωFQ endows the atoms of the nanostructure with a charge. In the presence of an external monochromatic electric field **E**^ext^(ω), the charge can be exchanged with nearest neighbor atoms as a result of Drude conduction^69^ and quantum tunnelling effect.^63^ For a system composed of *N* atoms, charges **q** can be calculated by solving the following set of linear equations:^68^
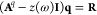
1where **A**^*q*^ is a real nonsymmetric matrix, which reads:
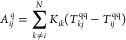
2**T**^qq^ is the charge–charge interaction matrix,^70^ while **K** is defined as
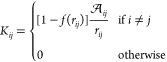
3Quantum tunneling effects are taken into account by means of the Fermi damping function *f*(*r*_*ij*_), which exponentially decays as a function of the interatomic distance *r*_*ij*_ = |**r**_*i*_ – **r**_*j*_| (**r**_*i*_ is the position of the *i*-th atom).  is an effective area connecting atoms *i* and *j*. Its value is based on the geometry of the system and has been chosen to best reproduce reference ab initio values.^61,63,64^ It is worth noticing that the **A**^*q*^ matrix only depends on the geometry of the ωFQ system, because it is a function of the interatomic distance *r*_*ij*_ only.

The frequency-dependent complex-valued factor *z*(ω) in eq 1 is defined as
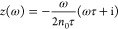
4where *n*_0_ is the electron density of the system and τ is the scattering time. The electron density depends on the composition and the morphology of the nanostructure. In general, for 3D systems  where σ_0_ is the static conductance of the material and m* is the effective electron mass, which for metallic systems is usually approximated to 1.^71^ For graphene-based structures, this approximation is no longer valid. Therefore, the electronic density can be written as^64,72^
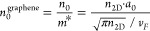
5
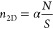
6where *n*_2D_ is the 2D numeral electronic density of the system, *a*_0_ is the Bohr radius, *v*_*F*_ is the Fermi velocity, *S* is the total surface of the graphene system, and α is the fraction of doping electrons per carbon atom. Such a number, and thus graphene plasmonic properties, can be tuned by varying the external gating, which is directly related to the Fermi energy (*E*_*F*_), which determines the numerical value of *n*_2D_ (and thus α) by
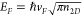
7Finally, the right-hand side in eq 1 is defined as^68^
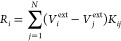
8where *V*_*i*_^ext^ is the electric potential associated with the external oscillating field evaluated at position **r**_*i*_, which implies we are assuming the quasistatic approximation to full EM equations.^63^

ωFQ has successfully been applied to the simulation of the plasmonic response of sodium nanostructures and graphene-based materials.^63−65^ However, the underlying Drude conduction mechanism is not able to reproduce the plasmonic response of *d*-metals, as for instance silver and gold nanoparticles, because interband (IB) transitions play an essential role.^73−77^ ωFQFμ correctly models such effects.^61^ There, each atom is endowed with both an oscillating charge *q*_*i*_ and an oscillating dipole **μ**_*i*_. The plasmonic response is then assumed to originate from two different mechanisms: the Drude conduction, taken into account by the charges, and the aforementioned IB transitions, which are treated by means of the dipoles, which account for the polarizability of the *d*-shell. By taking into account both terms, and their interaction, the plasmonic response of metal nanostructures, made by Ag/Au, could be correctly described.^61^ The resulting ωFQFμ equation reads:
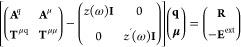
9where **A**^*q*^, *z*(ω), and **R** have been already introduced by eq 1. **T**^μq^ and **T**^μμ^ are the dipole–charge and dipole–dipole interaction tensors,^70^ whereas **A**^μ^ and *z*′(ω) are defined as follows:
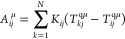
10
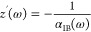
11where α_IB_(ω) is the IB polarizability, which is extracted from the experimental permittivity function (after removing the Drude part, see ref (61) for further details). Similarly to ωFQ, all the frequency-dependent terms are collected into a diagonal shift through the *z*, *z*′ functions, and the other terms depend on the geometry of the system only.

### 2.2 Coupling to a QM Hamiltonian

ωFQ and ωFQFμ can be coupled to a QM description of a molecular system in a QM/MM fashion.^78−85^ ωFQ and ωFQFμ describe the response to an external oscillating electric field. Thus, they can naturally be translated into a linear response formalism. However, to achieve a physically consistent description of the molecule/substrate system, their interaction needs to be modeled also in the ground state (GS). To this end, the analogous frequency independent force fields, FQ and FQFμ,^70,86−93^ can be exploited. In the following, we first briefly recall FQ and FQFμ for the GS. Then, we present the linear response formalism for the novel QM/ωFQ(Fμ) approaches.

#### 2.2.1 Description of the Ground State

The total energy of a two-layer QM/FQ(Fμ) system can be written as
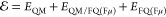
12where *E*_QM_ and *E*_FQ(Fμ)_ are the self-energies of the QM and FQ(Fμ) portions, whereas *E*_QM/FQ(Fμ)_ indicates their interaction energy. The latter term is described as the electrostatic interaction energy between the QM charge distribution and the classical fluctuating multipoles of the FQ or FQFμ force fields:^70^
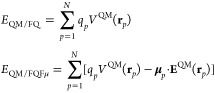
13where the sums run over MM atoms, *q*_*p*_ and **μ**_*p*_ indicate the *p*-th FQ and Fμ, which are located at position **r**_*p*_, while *V*^QM^ and **E**^QM^ are the electric potential and field generated by the QM portion, respectively.

If we exploit a DFT description of the QM portion, the ground state density can be determined by means of the effective Kohn–Sham (KS) equations, which are expressed in terms of the KS operator *h*_KS_. In this work, we use the current implementation of QM/FQ and QM/FQFμ in the Amsterdam Density Functional (ADF) module^94^ of the Amsterdam Modeling Suite (AMS) software package.^95^ There, the solution of KS equations is performed through a numerical integration scheme, in which the KS operator is built over a grid of points **r** in the molecular space of the QM portion, i.e.:
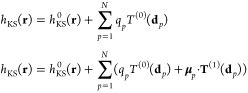
14In eq 14*h*_KS_^0^(**r**) indicates the KS operator associated with the isolated QM system, and the QM/FQ and QM/Fμ interaction tensors are introduced. They are defined in terms of the distance **d**_*p*_ between the *p*-th atom and the grid point (**d**_*p*_ = **r** – **r**_*p*_) as follows:
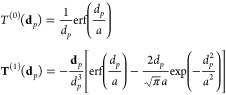
15where *d*_*p*_ = |**d**_*p*_| and *a* = 0.2 a.u.^96^ Since the molecular grid is constructed independently of the MM portion, in order to avoid numerical instabilities in eq 15, a screened interaction tensor between the QM grid points and the MM positions is considered, by analogy to what is reported in ref (97).

#### 2.2.2 Linear Response Theory and SERS Spectra

By following the approach reported in ref (35), SERS spectral intensities can be evaluated through the frequency-dependent complex polarizability tensor . To this end, the first-order variation of the molecular density under the effect of a time-dependent perturbation is required, which can be accessed by means of the linear response theory. In particular, as a perturbation, we consider a monochromatic uniform electric field **E**^ext^(ω), linearly polarized along the direction α = *x*, *y*, *z*. The perturbation operator, which acts on the electronic density, can be written as
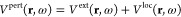
16where *V*^ext^ is the electric potential associated with the external field **E**^ext^ and *V*^loc^ is the local field operator, which takes into account the electric field generated by the plasmonic substrate (PS) as induced by the external field.^38^ Within ωFQ(Fμ), the local field operator reads:
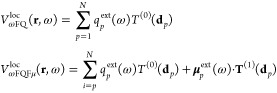
17*q*_*p*_^ext^(ω) and **μ**_*p*_^ext^(ω) can be calculated from eqs 1 and 9 by using *V*^ext^ as the source potential. In the following, we use the common notation for identifying the molecular orbitals (MOs): indices *i*, *j* for the occupied, *a*, *b* for the virtual and *r*, *s*, *t*, *u* for general MOs; moreover, given a quantity *X* we will indicate its variation at the first order with respect to the external electric field component α with *X*^α^. Given that, the first order density ρ^α^(**r**, ω) can be written as
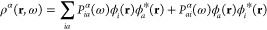
18where *P*_*rs*_^α^(ω) is the first-order density matrix expressed on the basis of the KS MOs. The nonzero elements of **P**^α^(ω) are the off-diagonal ones, which are associated with the occupied-virtual and virtual-occupied MOs. They can be computed through the Time-Dependent Kohn–Sham (TDKS) equations, which read:^98^
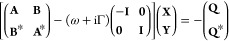
19where *X*_*ia*_ = *P*_*ia*_^α^ and *Y*_*ia*_ = *P*_*ai*_^α^. Also, the phenomenological damping factor Γ, which takes into account the finite lifetime of the QM excited state, is introduced.^99^ In addition, the following quantities are defined:
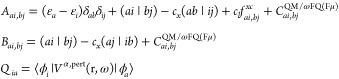
20where ε indicates MO energies, (*rs*|*tu*) two-electron integrals, and *c*_*x*_ and *c*_*l*_ define whether a pure (*c*_*x*_ = 0) or hybrid (*c*_*x*_ ≠ 0) DFT functional is exploited. Two additional contributions to TDKS equations arise for QM/ωFQ and QM/ωFQFμ: the local field in the right-hand side (see the definition of ***Q***, and eq 17), and the so-called image field or direct contribution to the left-hand side (***C***^*QM*/ωFQ(Fμ)^).^82^ The latter is also introduced in the context of polarizable embedding, such as FQ and FQFμ for nonabsorbing media, and determines the response of the MM variables to the perturbed density. Its expression for both QM/FQ and QM/FQFμ methods can be found elsewhere.^89,96,100^ On the other hand, the explicit contribution to the right-hand side is associated with the surface plasmon resonance and is responsible for the EM enhancement mechanism in surface-enhanced properties.

Once eq 19 is solved for the input frequency ω, the frequency-dependent polarizability tensor  is obtained as^101^
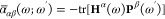
21where **H**^α^(ω) is the dipole matrix of the QM system, which involves both the dipole and the local field operator along the direction α, i.e.:
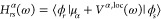
22From the physical point of view, the presence of the local field operator in eq 22 can be explained by the fact that the total scattered field from the molecule–nanostructures composite system contains two contributions: the scattered field from the molecule and the reflected field, which is generated by the molecule and reflected on the plasmonic nanostructure. In order to calculate Raman intensities, both fields need to be taken into account.^35,37^

Given the frequency-dependent polarizability tensor **α̅**(ω), Raman intensities can finally be evaluated by resorting to Placzek’s theory of Raman scattering.^102,103^ By assuming the perturbation field to be linearly perpendicular-plane polarized and the scattered light to be collected perpendicularly to the incident direction, the Raman intensity associated with the *k*-th normal mode can be calculated as^104^
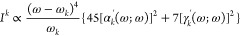
23where ω and ω_*k*_ are the frequencies of the external field and of the *k*-th normal mode, respectively, while α′ and γ′ are the isotropic and anisotropic polarizability derivatives with respect to the *k*-th normal mode coordinate *Q*_*k*_, i.e.:
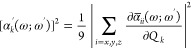
24
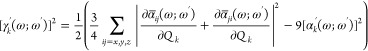
25The functional form of eq 23 has been obtained under the assumption that the frequency difference between the incident and scattered light is negligible.^34,104,105^ In the general case, the Raman intensity can be calculated as
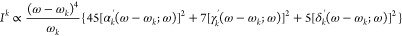
26in which [δ′(ω;ω′)]^2^ is the square of the antisymmetric anisotropy of the polarizability tensor derivative, i.e.:^41^
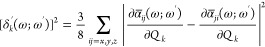
27In the case in which the incident and scattered frequencies are the same, the latter term is exactly zero and the Raman intensity is reduced to the one reported in eq 23. If the difference between the frequencies is large with respect to the broadness of the plasmonic absorption peak, this approximation may induce some differences in the final computed SERS intensity. In this work, similarly to other approaches,^34,35^ we have relied on this approximation, and its implications will be topic of future communications.

## 3 Computational Details

Silver and gold NPs’ geometries are constructed by using OpenMD.^106^ In particular, cuboctahedron (cTO), icosahedron (Ih), and ino-decahedron (i-Dh) morphologies are considered. Graphene disks’ (GDs) geometries are generated by using the VMD package^107^ by cutting a graphene sheet in a circular shape with radius *r* and removing dangling bonds, which only marginally affect GDs’ plasmonic response.^68^ For both metal NPs and GDs, we study SERS signals as a function of the PS size. To this end, we consider GDs with radius 20 ≤ *r* ≤ 160 Å and metal NPs composed of a maximum of 10179 atoms (see also Tables S2 and S3 in the Supporting Information (SI)). The dipolar plasmon resonance frequency (PRF) of each PS (see Tables S2 and S3 in the SI) varies from 3.64 to 3.42 eV for Ag, and from 2.31 to 2.17 eV for Au. In the case of GDs, the PRF varies from 0.61 to 0.24 eV. In QM/ωFQ(Fμ) calculations, pyridine (PY) is described at the QM level by using BP86 or B3LYP DFT functionals, as coupled with a double−ζ polarized (DZP) or a triple−ζ polarized (TZP) basis set.^108^ To simulate Raman/SERS spectra, frequency-dependent polarizabilities α̅(ω) are calculated^109,110^ by setting Γ (see eq 19) to 0.10 eV.^35^ The α̅(ω) geometrical derivatives are obtained by means of a numerical differentiation scheme^111−113^ by using a constant step size of 0.001 Å. Similar results are obtained by using a step size of 0.0005 Å (see Figure S1 in the SI). In this first application, normal modes of displacement are calculated on the isolated QM molecule because we expect vibrational frequency shifts induced by the nanostructure to be negligible, as has been shown by previous studies (see for instance refs (35), (40), and (105)). Frequency-dependent polarizabilities and SERS spectra are calculated by setting the frequency ω as the PRF of each plasmonic substrate (see Tables S2 and S3 in the SI); moreover, the final SERS spectra are obtained by convoluting raw data with Lorentzian band-shapes (full width at half-maximum (fwhm) of 4 cm^–1^). The ωFQ parameters for GDs are taken from ref (64), whereas ωFQFμ parameters for silver and gold are taken from ref (61). All QM/ωFQ and QM/ωFQFμ calculations are performed by using a locally modified version of the AMS software.^94,95^

In the following, we evaluate the Raman enhancement associated with the *k*-th normal mode in terms of the enhancement factor (EF):
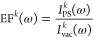
28where *I*_PS_^*k*^(ω) and *I*_vac_^*k*^(ω) are the Raman intensities of the *k*-th normal mode evaluated for the molecule-PS and the molecule in gas-phase systems, respectively (see eq 23). Since EF clearly depends on the selected normal mode, it is convenient to define a spectrally averaged enhancement factor (AEF) as follows:^49^
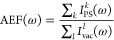
29where the indices *k* and *l* run over the normal modes of the target molecule (in the selected spectral region). As an additional measurement of calculated enhancement, we also introduce the maximum enhancement factor (MEF), i.e.:
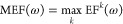
30

## 4 Numerical Results

**Figure 1:**
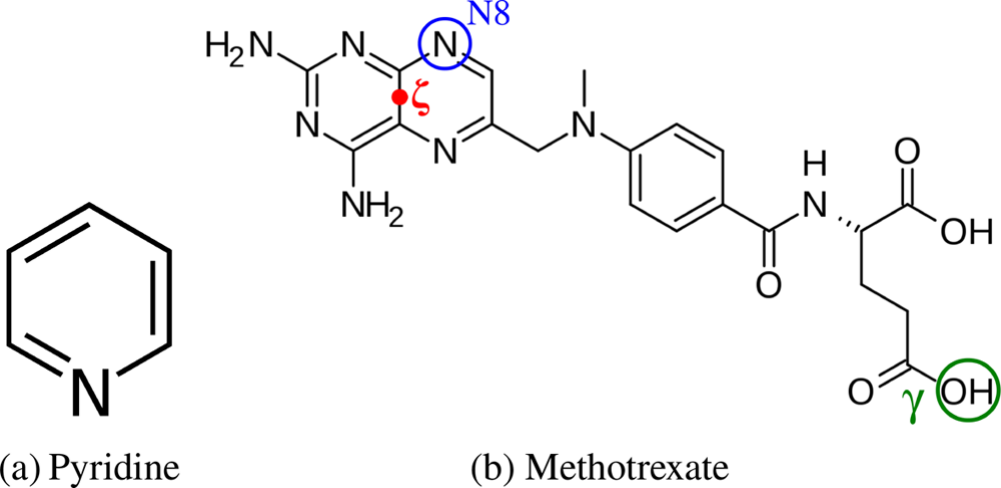
Pyridine (a) and methotrexate (b) molecular structures. In panel b, we highlight the position ζ (red), which indicates the center of MTX aromatic rings, the N8 atom (blue) and the OH (green) of the γ-carboxyl group.

In this section, we first discuss the capability of QM/ωFQ and QM/ωFQFμ to correctly describe the physicochemical features of the molecule-plasmonic substrates system. To this end, QM/ωFQ and QM/ωFQFμ approaches are first applied to the simulation of SERS spectra of pyridine (PY, see Figure 1a), which has been the first molecular system for which SERS was experimentally observed.^1,18,114^ Also, thanks to its small size, PY is a perfect prototype to test our novel approach. PY is adsorbed on two PSs: metal NPs and graphene disks (GDs). We first validate our novel approach by studying the dependence of Raman enhancements on the morphology and the PS chemical composition, the molecule-PS distance, and spatial arrangement. Finally, we present an application of the approach to the simulation of a real-case scenario, i.e. methotrexate (MTX, see Figure 1b), adsorbed on a graphene disk.

### 4.1 Model Testing

#### 4.1.1 Metal Nanoparticles

##### 4.1.1.1 Dependence of Enhancement Factors on the Nanostructure Properties

**Figure 2:**
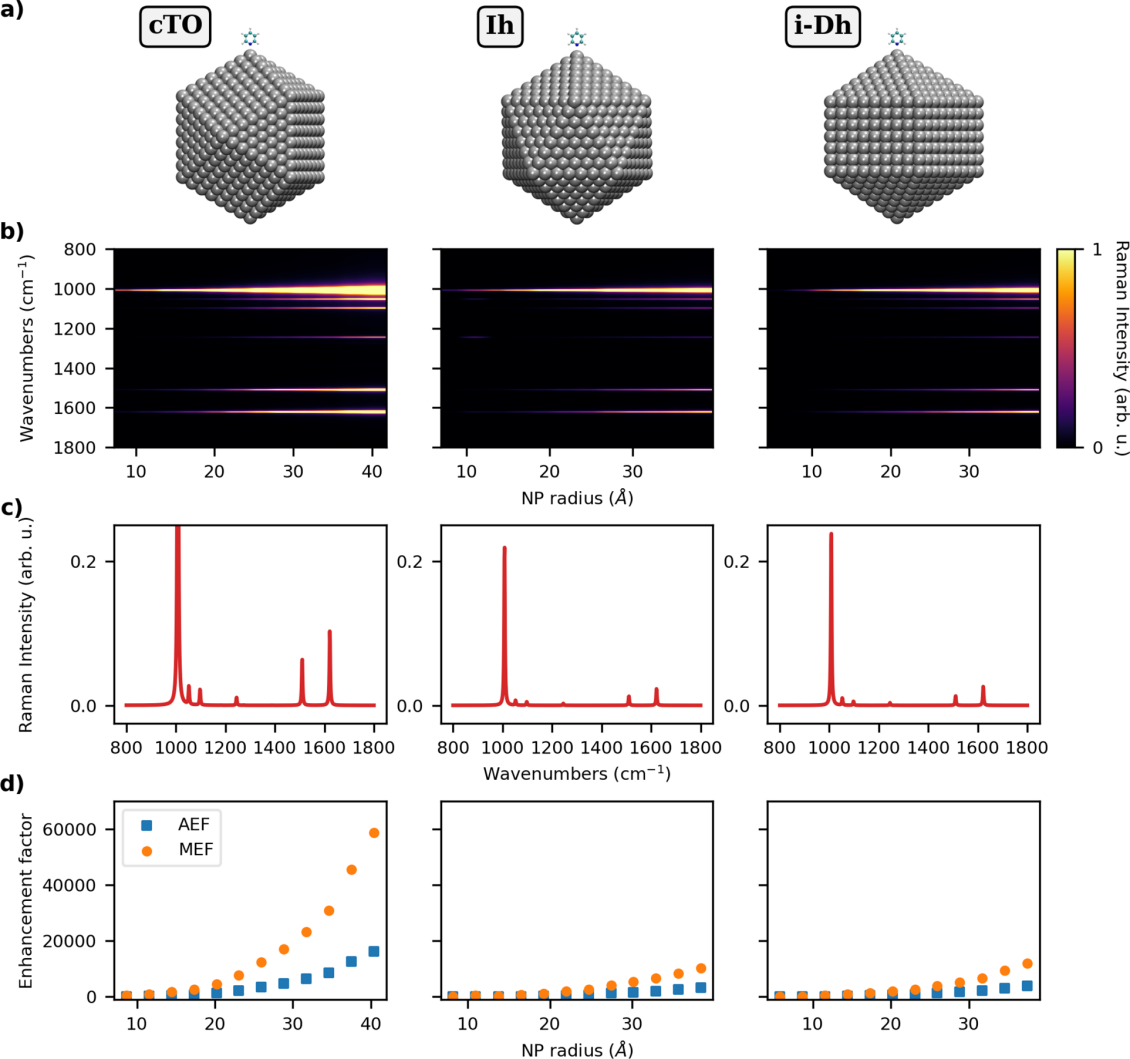
a) Graphical depiction of PY-Ag cTO (left), Ih (middle), and i-Dh (right) systems; (b) color plot of normalized SERS spectra as a function of the NP radius; (c) normalized SERS spectra of PY adsorbed on the largest NP structure for each shape; (d) AEF and MEF as a function of the NP radius. SERS signal is computed at the PRF of each Ag NP (3.54–3.42 eV for cTO; 3.64–3.51 eV for Ih; 3.52–3.42 eV for i-Dh; see also Table S2 in the SI).

**Figure 3:**
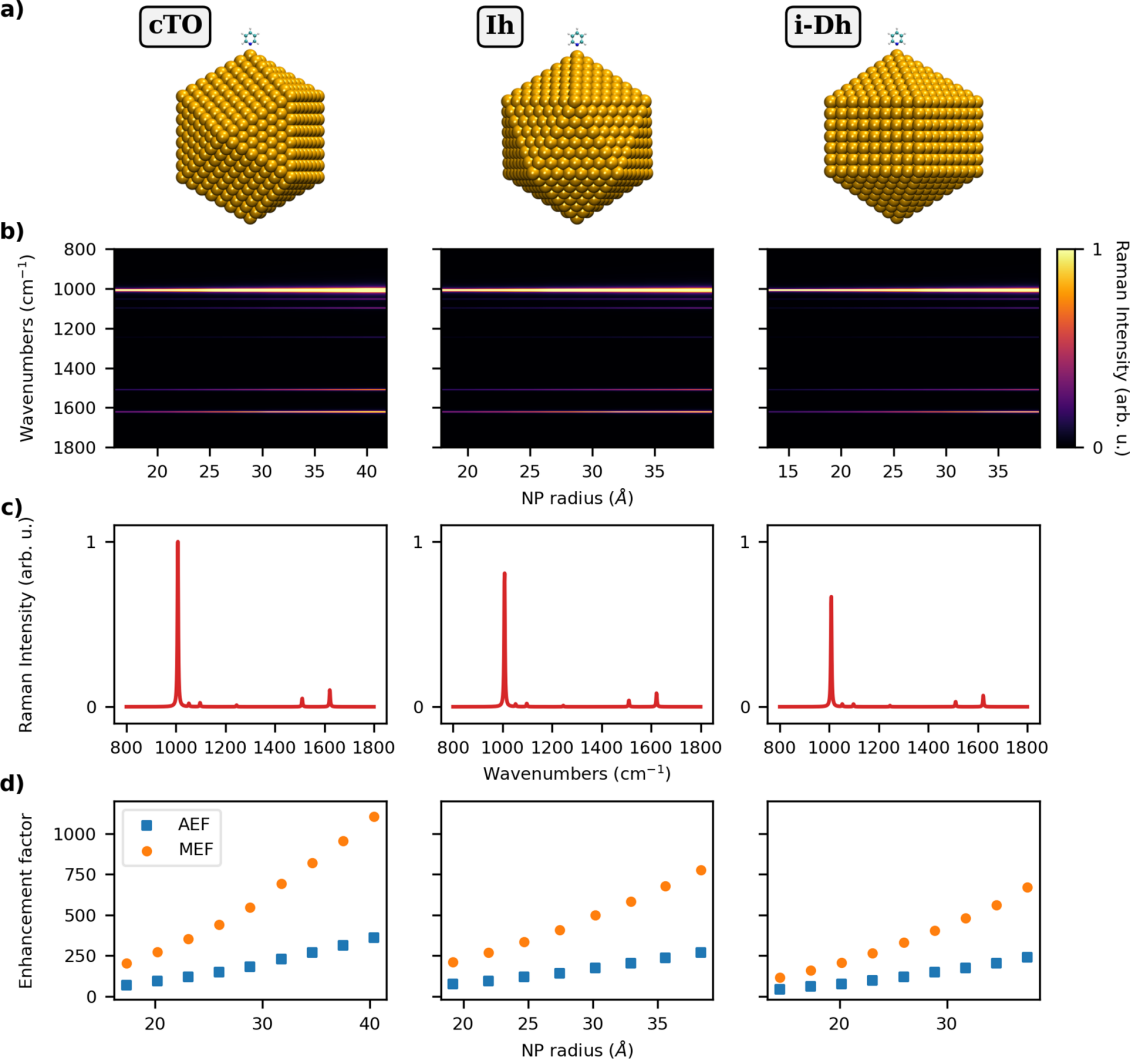
a) Graphical depiction of PY-Au cTO (left), Ih (middle), and i-Dh (right) systems; (b) color plot of normalized SERS spectra as a function of the NP radius (Å); (c) normalized SERS spectra of PY adsorbed on the largest NP structure for each shape; (d) AEF and MEF as a function of the NP radius. SERS signal is computed at the PRF of each Au NP (2.27–2.17 eV for cTO; 2.29–2.21 eV for Ih; 2.31–2.17 eV for i-Dh; see also Table S2 in the SI).

In this section, we study how the morphology, the size, and the chemical composition of the PS affect the SERS signal. To this end, PY is adsorbed on silver and gold cTO, Ih, and i-Dh NPs with a radius varying from about 6 to 40 Å (see Figures 2a and 3a and Table S2 in the SI). PY is adsorbed standing on the vertex of each structure along the *y* axis by setting a distance of 3 Å between the Nitrogen atom and the NP vertex, similarly to previous studies.^35^ The results obtained by treating PY at the B3LYP/DZP level of theory are graphically reported in Figures 2 and 3, for Ag and Au NPs, respectively (see Figure S2 in the SI for their analogous calculated at different levels of theory). In particular, in Figures 2b and 3b SERS spectra of the three different configurations as a function of the NP radii are reported, whereas in the corresponding c panels, the dependence of both AEF and MEF on the NP radii is graphically depicted. Note that the introduction of such a screening function guarantees the stability of the results by changing the DFT integration grid (see Figure S3 in the SI).

Let us first discuss the results obtained for Ag NPs. By focusing on Figure 2b, it can be noticed that SERS intensities strongly depend on the NP size, increasing as the NP size increases. The enhancement is not the same for all normal modes. Indeed, the vibrations modulating the components of the electronic polarizability orthogonal to the NP surface are associated with larger SERS intensity, because both the incident radiation and the scattered field benefit from the EM enhancement. For instance, this is evident for the ring breathing mode at 1008 cm^–1^ (see Figure S4e in the SI) and the symmetric bending modes of the α-hydrogen atoms at 1510 and 1622 cm^–1^ (see Figure S4n,p in the SI), which are characterized by the largest SERS intensities (see also Figure 2c where SERS spectra for the largest NPs are graphically reported). It is also interesting to note that depending on the morphology of the NPs, a different SERS spectrum can be obtained. In fact, Figure 2b shows that SERS spectra obtained by adsorbing PY on Ag Ih and i-Dh NPs display a significantly different relative intensity patterns as compared to PY on Ag cTO SERS spectrum. This is important to remark, as it shows that the details of the local electric field distribution (such as electric field gradients) are accounted for in the modeling and have a visible effect on SERS spectra. A selection of all enhancement factors of PY adsorbed on selected PS is reported in Table S4 in the SI.

**Figure 4:**
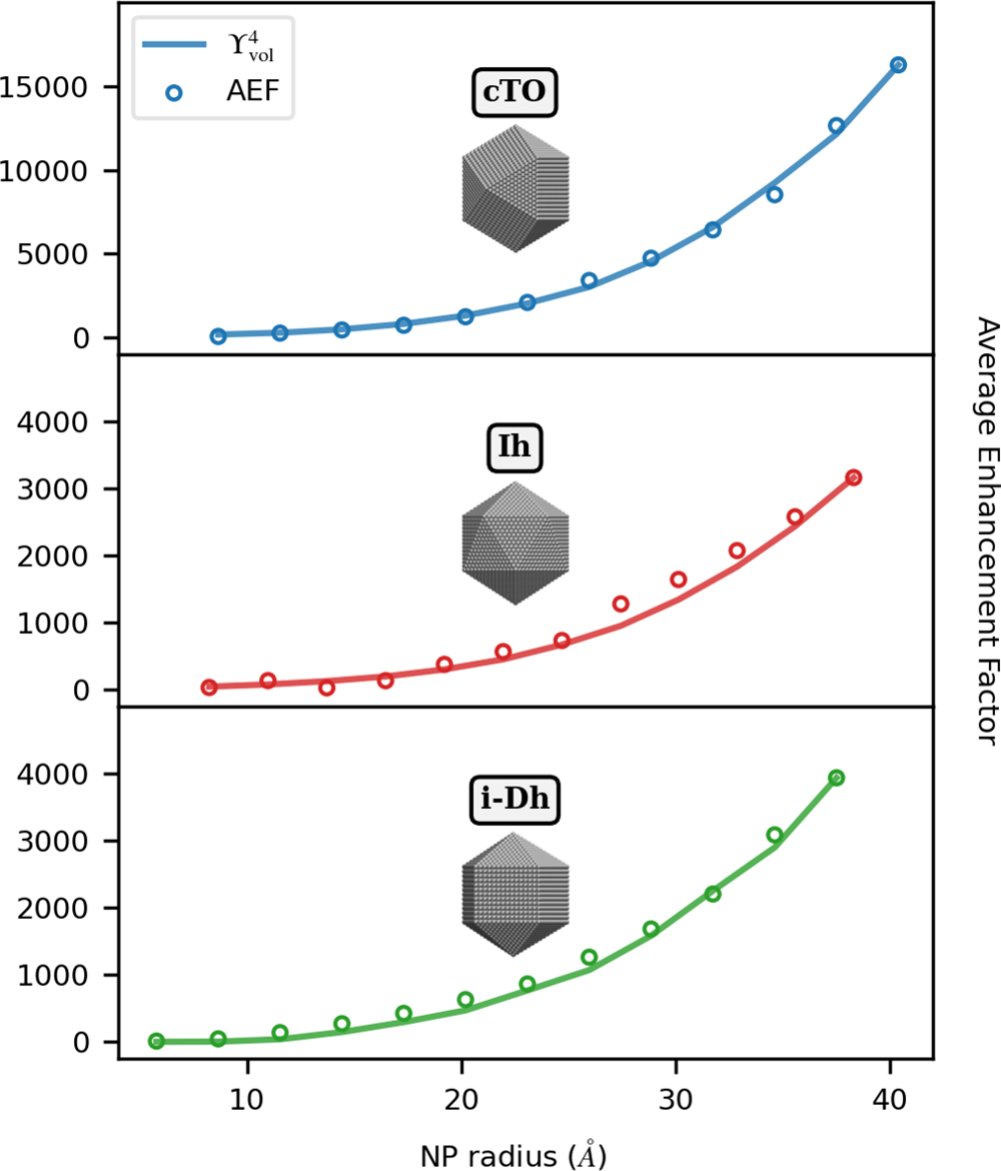
AEF (circles) and normalized Υ_vol_^4^ (solid line, see eq 31) for PY-Ag as a function of the NP radius and morphology (cTO, Ih, and i-Dh). The SERS signal is computed at the PRF of each Ag NP (3.54–3.42 eV for cTO; 3.64–3.51 eV for Ih; 3.52–3.42 eV for i-Dh; see also Table S2 in the SI).

To further analyze such results, Raman enhancements can be quantified in terms of AEF and MEF (see eqs 29 and 30). Their dependence on the NP radius is reported in Figure 2d, which shows that the shape of the NP strongly affects their values. In particular, cTO-based structures yield the largest enhancement factors as compared to the other considered morphologies. Interestingly, for small nanostructures, the most enhanced normal mode is the asymmetric bending of α-hydrogen atoms at 1388 cm^–1^ (see Figure S4l in the SI), while for the largest structures the MEF is associated with the symmetric bending of the same α-hydrogen atoms at 1510 cm^–1^ (see Figure S4n in the SI). In order to rationalize the differences between the three different shapes, we can resort to the so-called *E*^4^ approximation,^15,115^ which states that Raman enhancements are proportional to the fourth power of the electric field induced on the NP. Thus, we define Υ_vol_^4^ as follows:
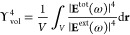
31where *V* is the molecular volume, **E**^tot^(ω) is the total electric field, whereas **E**^ext^ is the incident external electric field aligned with NP main axis. **E**^tot^(ω) is computed on a box, with sides placed at a distance of 1 Å from each PY atom. It is worth remarking that in ωFQFμ the fluctuating multipoles are associated with a spherical Gaussian distribution,^61,68^ which is taken into account in the calculation of the electric field intensity.^60^ The AEF-Υ_vol_^4^ correlation as a function of the NP radius is depicted in Figure 4 for Ag cTO, Ih, and i-Dh substrates. Note that Υ_vol_^4^ values are normalized to the largest AEF for each morphology. Absolute AEF and Υ_vol_^4^ values are reported in Table S4 in the SI. The two data sets quantitatively differ, probably due to the high inhomogeneity of the electric field in the proximity of the NP surface, in agreement with ref (35).

The agreement between AEF and Υ_vol_^4^ is almost perfect, independently of the size and shape of the Ag NPs. Also, we can justify the larger enhancement factors (AEF and MEF) reported in Figure 2c for cTO with respect to Ih and i-Dh arrangements. Indeed, they are due to a greater induced field, as shown in Figure 4. This is not surprising and is in line with the results recently reported by us in ref (61), which highlighted cTO as the most effective morphology to provide near-field enhancement.

A similar analysis can also be performed by modifying the chemical composition of the NPs. To this end, we consider the same NPs shapes, but made of Au atoms (see Figure 3a). As recently reported in ref (61), ωFQFμ predicts a dipolar PRF only for Au NPs with a radius larger than 15 Å (see also Table S2 in the SI). SERS spectra and the AEF/MEF of PY adsorbed on Au cTO, Ih, and i-Dh arrangements as a function of the radius are reported in Figure 3b,c,d, respectively.

Similarly to the Ag case, the most intense SERS signals are associated with the normal modes involving the PY ring (1008 cm^–1^, see Figure S4e in the SI) and the α-hydrogen atoms (1510 and 1622 cm^–1^, see Figure S4n,p in the SI, respectively). However, the computed SERS intensities are much lower as compared to the PY-Ag case. By inspecting SERS spectra calculated by considering the largest Au NP (see Figure 3c), we can indeed notice that slight qualitative discrepancies are reported with respect to PY-Ag (see Figure 2c). In fact, the SERS spectra of PY on the selected morphologies are characterized by similar relative intensities between the most intense peaks, and the differences among the three spectra are thus less accentuated than the PY-Ag case.

Computed AEF/MEF indices (see Figure 3d) are also much lower as compared to the PY-Ag case, independent of the NP shape. In this regard, it is worth highlighting that Au PRF is lower than Ag PRF (∼2.2 eV vs ∼3.5 eV), and this also numerically affects the Raman intensities of the molecule in the gas phase. However, the noticeable decrease of AEF/MEF is primarily due to the fact that the electric field generated by the dipolar plasmon of Au NP is much less intense than for Ag. Therefore, a smaller enhancement of the Raman intensities is observed. This is demonstrated by the numerical values of AEF, and by the fact that the correlation between AEF and Υ_vol_^4^ worsens (see Figure S5 in the SI). The reported behavior of Au nanoparticles (see Figure S5 in the SI) has also been previously reported by exploiting other QM/classical approaches.^35^

In case of Au, the *E*^4^ approximation is less accurate, probably because the local field is less intense. In fact, AEF results from the mixing of vibrational normal modes that are enhanced according to different powers of the electric field, up to *E*^4^. When the local field experienced by the molecular system is large, the *E*^4^-dependent vibrational normal modes will dominate the AEF value, thus clearly complying with the *E*^4^ approximation (see Section S1 in the SI for further details).

##### 4.1.1.2 Dependence of AEF on PY-NP Distance

**Figure 5:**
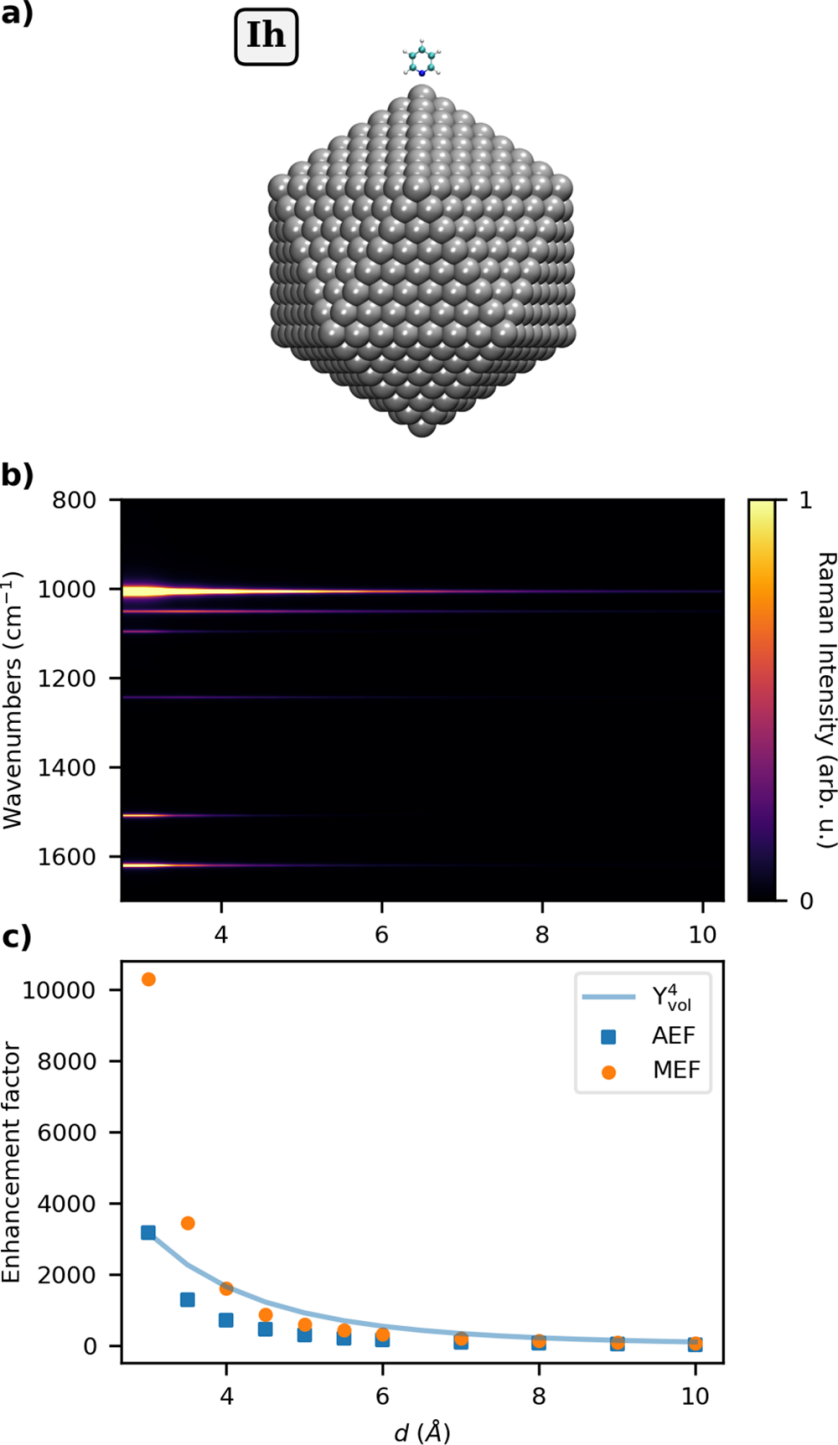
(a) Graphical depiction of PY-Ag_10179_ Ih system; (b) color plot of normalized SERS spectra as a function of the PY-NP distance *d* (Å); (c) AEF, MEF, and normalized Υ_vol_^4^ as a function of *d*. The SERS signal is computed at the PRF of the Ag NP (3.51 eV, see also Table S2 in the SI).

As it has been stated in section 2.2, the local field plays a key role in the enhancement of Raman intensities. In this section, we study how AEF behaves as a function of the distance between the molecule and the PS. As a proof of concept, we consider again the case of PY adsorbed on the largest Ih Ag NP (10179 atoms, radius = 38.30 Å, see Table S2 in the SI), for which we compute the SERS spectrum by increasing the distance *d* between the Nitrogen atom and the PS tip. QM/ωFQFμ results are reported in Figure 5 for 3 ≤ *d* ≤ 10 Å. Analogous results for Au NP are given in Figure S6 of the SI.

As expected, Figure 5 clearly shows that Raman intensities rapidly decay by increasing *d* (∼*d*^–4^). In particular, AEF decreases from about 1000 at *d* = 3 Å to about 14 at *d* = 10 Å. A similar behavior is observed for MEF. In particular, for *d* < 4 Å, MEF is associated with the symmetric bending of the α-H (1510 cm^–1^, see Figure S4n in the SI), while for *d* ≥ 4 Å the asymmetric bending of the same hydrogens (1098 cm^–1^, see Figure S4h in the SI) yields the maximum enhancement.

To rationalize these findings, AEF values as a function of *d* can be compared to the aforementioned *E*^4^ approximation. To this end, we compute the induced electric field in the molecular PY volume as a function of the PY-PS distance. Such values are graphically depicted in Figure 5c (see Figure S6c in the SI for the Au NP case). As also commented above for Figure 4, both the approximated estimation Υ_vol_^4^ and computed AEF rapidly vanish as the distance increases. Indeed, it can be noticed that the curves follow a different trend as a function of the distance, which can be ascribed to the fact that AEF comes from the average of different enhancement factors associated with vibrational normal modes according to different powers of the electric field, up to *E*^4^. Also for Au (see Figure S6c in the SI), small discrepancies are reported, probably related to lower local field effects compared to Ag. Absolute AEF and Υ_vol_^4^ values are reported in Table S7 in the SI.

##### 4.1.1.3 Dependence of AEF on Molecule-PS Configuration

**Figure 6:**
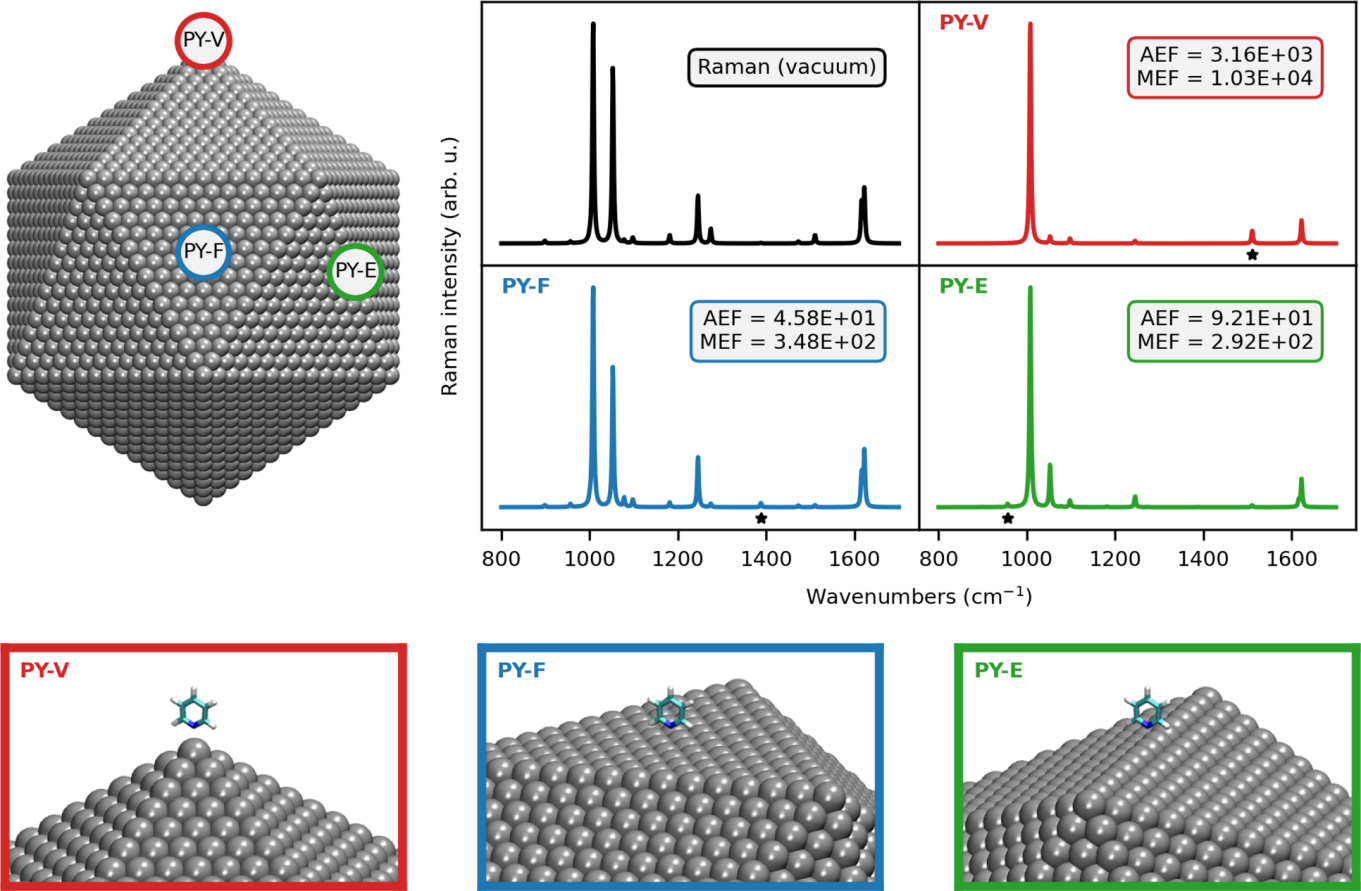
Normalized QM/ωFQFμ PY SERS spectrum as a function of the adsorption site of PY on the Ih Ag_10179_ NP (upper left and bottom panels). AEF and MEF for each configuration are also given. The vibrations associated with the MEF are indicated with a star. The SERS signal is computed at the PRF of the Ag NP (3.51 eV, see also Table S2 in the SI).

In the previous examples, we have discussed how SERS spectra might depend on the NP morphology and chemical nature of the atoms constituting the NP, as well as on the mutual molecule-NP distance. In this section, we discuss how the relative configuration of the molecule-PS system affect the SERS spectrum and enhancement. As a proof of concept, we consider three different PY adsorption positions on Ag_10179_ Ih NP. The most representative points of the icosahedral structure are selected (see Figure 6, bottom): vertex (PY-V, red dot), edge (PY-E, green dot), and face (PY-F, blue dot). In all configurations, PY is adsorbed perpendicularly to the NP surface at a distance of 3 Å, with the nitrogen atom laying closest to the NP. As for the previous cases, the Raman signal is computed by means of eq 23, by irradiating the PY/Ag_10179_ system with an external electric field polarized along the *x*, *y*, and *z* directions. Calculated QM/ωFQFμ SERS spectra are graphically reported in Figure 6, together with the computed Raman spectrum of PY in the gas phase.

As a result of the PY-NP interaction, the Raman spectrum undergoes drastic changes when moving from the in vacuo to the adsorbed case. This can be particularly appreciated by the changes in relative intensities between the two most intense Raman peaks in vacuo, which are associated with the ring breathing (1008 cm^–1^, see Figure S4e in the SI) and the symmetric bending of the α-H (1053 cm^–1^, see Figure S4f in the SI), respectively. Indeed, for all the selected absorption sites, the relative intensity of the bending mode (1053 cm^–1^) decreases with respect to that of the ring breathing mode (1008 cm^–1^), almost vanishing in the case of PY-V (red). Other major differences for PY-F (blue), PY-E (green), and PY-V (red) are reported, for which the relative intensity between the most intense peaks (1000–1100 cm^–1^) and the other dominant bands completely differ with respect to the gas phase.

The spatial PY-PS arrangements not only affect the spectral shape but also the enhancement factors. In this respect, AEF for PY-V is 2 orders of magnitude larger than the values computed for both PY-F and PY-E configurations. The same trend is also observed for MEF, which is interestingly associated with different normal modes depending on the relative PY-PS position. In fact, normal modes involving α-H atoms feature the maximum enhancements for all cases (graphically highlighted in Figure 6 with a star): symmetric bending (PY-V case, 1588 cm^–1^, Figure S4n in the SI), asymmetric bending (PY-F case, 1388 cm^–1^, Figure S4l in the SI), and out-of-plane vibrations (PY-E, 956 cm^–1^, Figure S4b in the SI). Such differences are directly related to the relative PY-PS arrangements and are intuitively associated with normal modes providing the largest polarizability variation.

All the reported differences, in both computed SERS spectra and AEF/MEF values, can be related to the inhomogeneity of the electric field induced by the geometrical shape of the Ag NP, which clearly differs by moving for the vertex (PY-V) to a face (PY-F) of the Ih morphology. In fact, the largest changes with respect to the gas-phase spectra/values are predicted for the PY-V configuration (red), for which we observe the largest AEF (∼10^3^) and a drastically different Raman spectrum. Such findings are perfectly explained by the tip effect,^69^ which characterizes most plasmonic materials. In fact, in the PY-V configuration, PY is adsorbed to the sharpest region of the Ih NP.

In order to show the effect of the inhomogeneity of the electric field in the surroundings of metal nanoparticles, we have performed additional calculations on the pyridine/Ag_10179_ system in the Ih morphology by changing the relative position of the molecular substrate with respect to the tip of the Ih nanostructure (see Figure S7 in the SI for more details). For each of these configurations, SERS spectra and AEF values have been computed, and the results are reported in Figures S7 and S8.

##### 4.1.1.4 Comparison with Available Experiments

**Figure 7:**
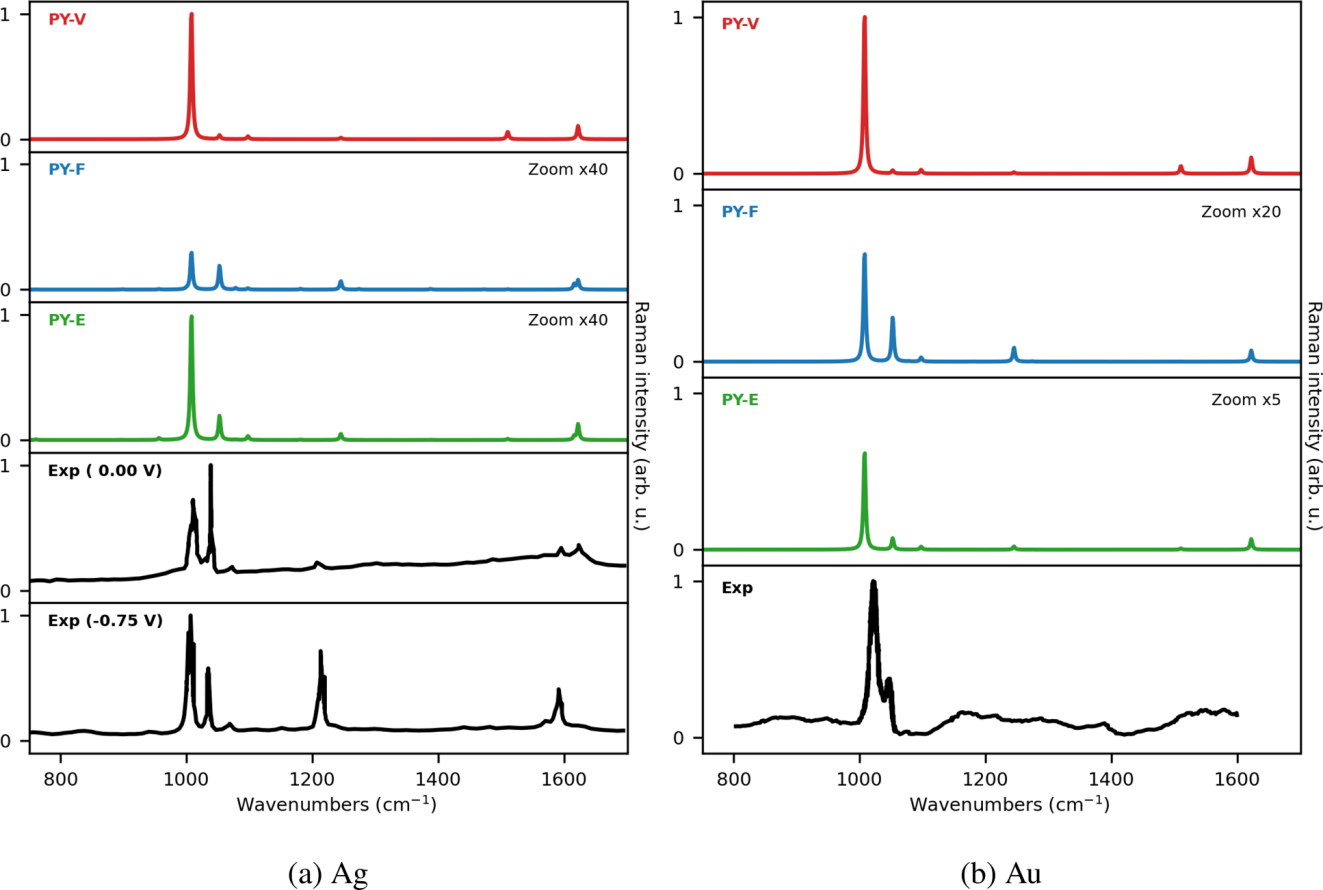
QM/ωFQFμ SERS spectra of PY adsorbed on Ag_10179_ (a) and Au_10179_ (b) Ih NPs (see also Figure 6). Experimental spectra measured for Ag (a, at 0.0 eV and −0.75 eV external bias) and Au (b, at 0.0 eV) electrodes, reproduced from ref (116). (Ag) and 117 (Au) are also reported. The SERS signal is computed at the PRF of the NPs (3.51 eV for Ag; 2.21 eV for Au; see also Table S2 in the SI).

To conclude the discussion, we compare our QM/ωFQFμ calculations with experimental data available in the literature (Figure 7 (a-Ag, b-Au).^116,117^ We note that our calculations are based on an ideal representation of experimental conditions. Instead, measured spectra result from the interplay between several effects which are not necessarily accounted for in the modeling, such as solvent effects, the coating of the metal electrode, external bias applied, impurities and the roughness of the metal surface. For these reasons, similarly to previous computational studies,^35^ the comparison between our results and experiments may only be qualitative.

Let us first focus on PY SERS on the Ag electrode, for which experimental spectra at different bias potential have been measured (see Figure 7).^116^ The experimental spectrum at zero bias (0.00 V) is dominated by two bands at about 1000 and 1050 cm^–1^, whose relative intensities are inverted at higher voltages (see bottom panels). Remarkably, the same bands are also the most intense in computed QM/ωFQFμ SERS spectra, for all investigated PY-PS configurations. Another relevant feature of experimental SERS spectra is the band at about 1200 cm^–1^, which only becomes visible by increasing the external bias (see bottom panels in Figure 7). For this reason, such a band has been commonly associated with a CT mechanism. As stated above, QM/ωFQFμ only accounts for the EM mechanism. Remarkably, and differently from previous models,^35^ our approach is able to predict such a feature, because the 1200 cm^–1^ peak is almost absent in the SERS spectrum of the most intense configuration (PY-V, top panel), thus confirming previous hypotheses assigning that band to CT effects.

Similar qualitative trends hold for the Au substrate. Indeed, the experimental SERS spectrum (which refers to an unbiased electrode) is dominated by the bands at about 1000–1050 cm^–1^. A similar behavior is observed in the computed spectrum. In addition, the band at about 1200 cm^–1^ is almost absent in both experimental and theoretical SERS spectra.

#### 4.1.2 Graphene Disks

**Figure 8:**
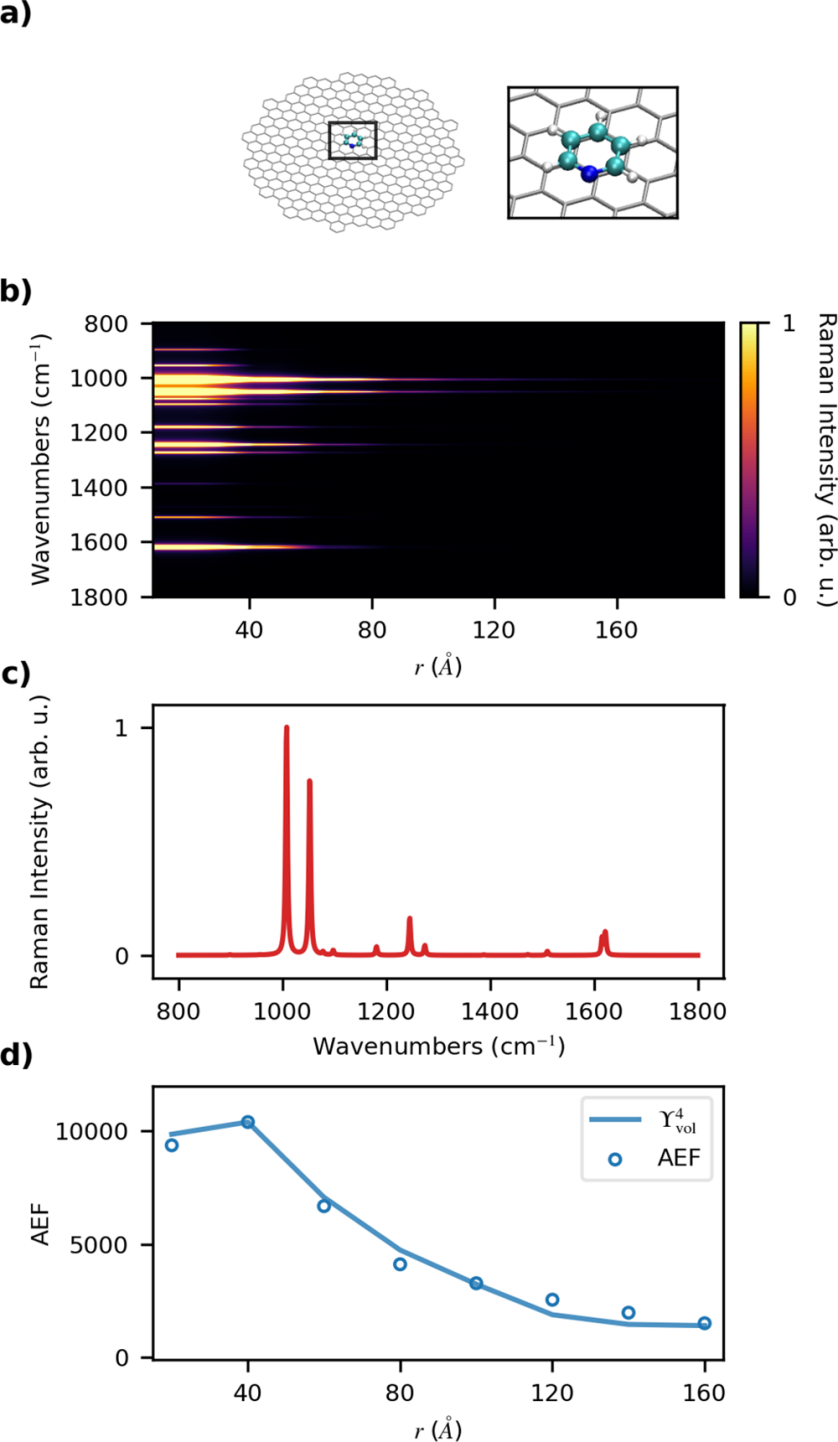
(a) Graphical depiction of the PY-GD system. (b) Color plot of normalized SERS spectra as a function of the GD radius *r* (Å). (c) Normalized SERS spectra of PY adsorbed on a GD with *r* = 40 Å, (d) AEF and Υ_vol_^4^ as a function of *r*. The SERS signal is computed at the PRF of each GD (0.61–0.24 eV, see also Table S3 in the SI).

In order to show the versatility of QM/ωFQ(Fμ), here we extend the previous study to PY adsorbed on 2D graphene-based substrates,^118,119^ which to this end, is a set of 8 GDs with a radius 20 < *r* < 160 Å (see also Table S3 in the SI). PY is assumed to be adsorbed on the GD center of mass, parallel to the GD surface at a distance of 3 Å (see Figure 8a). Such a distance is chosen because it is close to the equilibrium PY-GS distance reported in the literature.^120^ For each PY-GD, GERS spectra are calculated at the B3LYP/DZP level of theory, and the results are reported in Figure 8b. Note that, in all calculations, *E*_*F*_ = 0.4 eV, according to typical reported Fermi energy values.^121,122^ We note, however, that the PRF of graphene-based materials can be tuned by varying the external bias, thus affecting *E*_*F*_. When applied to GERS, such a feature can be exploited to make the PRF coincide with a molecular excitation of the system under investigation, thus resulting in a pragmatical method to yield resonance Raman assisted by graphene plasmons. Although our approach is general enough to account for such an effect, this is not investigated in this first work.

From the analysis of GERS spectra, we first note that Raman relative intensities are different from those reported in the case of PY-metal NP systems (see Figures 2 and 3), with the presence of additional peaks. The latter are mainly related to normal modes involving the vibrations of β and γ H atoms of the pyridine ring (1181 and 1274 cm^–1^, see Figure S4i,k in the SI). This is the result of the PY-GD morphology under investigation. In fact, the parallel configuration of the PY ring with respect to the GD plane allows for a large variation of the polarizability associated with the aforementioned normal modes. Nevertheless, GERS spectra are still dominated by the PY ring breathing and the asymmetric bending of the α hydrogens (1000–1100 cm^–1^), as already reported for the case of metal NPs. The most relevant dissimilarity between the two substrates is the dependence of GERS spectra on the GD *r*. In fact, Figure 8 clearly shows that for a large *r* (≥120 Å) the Raman signal vanishes as compared to for a small *r* (≤40 Å), thus reporting the opposite trend as compared to metal NPs (see Figures 2b and 3b).

To rationalize this trend, AEF values as a function of *r* are reported in Figure 8d. Absolute AEF and Υ_vol_^4^ values are given in Table S5 in the SI. Different from metal NPs, in this case the two data sets are almost in perfect agreement, even quantitatively. This is related to the absence of inhomogeneities in the induced field at the center of the graphene disk.

A local maximum is observed for *r* = 40 Å, which rapidly decays for larger *r* values. As for the metal NPs, the electric field generated by the GD increases with the radius. Therefore, a larger enhancement of the Raman intensity is expected. However, for the specific configuration of the GD system, the electric field is much more intense on the edges (see ref (60)). Since the distance between PY (adsorbed on the GD center of mass) and the GD edges increases with *r*, the enhancement factor and the Raman signal decrease as compared with small *r* (see Figure 8b). The combination of such effects is responsible for the local maximum in the 40 Å case, in which the electric field at the center of the GD is large enough to substantially increase Raman intensities and therefore PY enhancement factors. Indeed, the trend of AEF as a function of GD *r* can be explained by means of the Υ^4^ approximation, analogously to metal NPs. The correlation between AEF and Υ_vol_^4^ is shown by Figure 8d; the agreement is almost perfect, thus justifying the reported behavior in terms of EM enhancement.

We finally remark that our methodology can in principle simulate all possible PY-GD configurations. However, in this work we have restricted the analysis to the selected geometry because it better represents, from the physicochemical point of view, the most favorable configuration of a target molecule adsorbed on a graphene sheet, i.e. far from the edges. Within this picture, in fact, we aim to mimic the common experimental setup that is generally constituted of a graphene substrate with intrinsic dimensions much larger than those considered in this work.^67^ We point out that different PY-GD configurations can provide higher enhancements, as we have recently reported in ref (60).

### 4.2 Increasing the Chromophore Complexity: Methotrexate Adsorbed on Graphene Disks

In this section, we discuss the application of the model to a realistic case, i.e. methotrexate adsorbed on a graphene disk (see Figure 1b for the molecular structure). Differently from PY, MTX is a flexible molecule. Therefore, reliable modeling needs to account for the different conformations. To this end, we perform molecular dynamics (MD) simulations by using ReaxFF,^123,124^ within its dedicated engine^125^ in the Amsterdam Modeling Suite (AMS).^95^ The ReaxFF force field developed in ref (126), and which has been reported to reliably describe graphene-based systems, is employed.^127^ Temperature is maintained at 300 K by using a Nosé–Hoover chain (NHC) thermostat,^128^ with a damping constant of 100 fs. In NPT simulations, constant pressure is enforced by using the Martyna–Tobias–Klein barostat with a damping constant of 100 fs.^129^ 20 layers of graphite, composed of 50 atoms each, are prepared in a 3-D hexagonal periodic cell with lattice parameters *a* = *b* = 12.3 Å and *c* = 67.1 Å. A 100 ps NPT calculation is then run by imposing a pressure of 1 atm in all directions. At this point, the simulation box is enlarged so that *a* = *b* = 62.52 Å and *c* = 100 Å, and only 10 layers of graphite, formed by 1250 carbon atoms each, are kept. Then a 50 ps NPT simulation is performed by imposing a pressure of 1 atm in the planar directions. No substantial changes in the values of *a* and *b* are detected during pressure equilibration. After this, MTX is thermalized in an empty 3-D hexagonal periodic box with lattice parameters *a* = *b* = 62.52 Å and *c* = 100 Å by means of a 62.5 ps NVT simulation. Thermalized MTX is then placed at a distance of ∼10 Å from the equilibrated graphitic surface and a production NVT simulation of 375 ps is performed. After 125 ps, the temperature is equilibrated and the center of the aromatic rings of MTX (ζ-point in Figure 1b) is adsorbed at an average distance of ∼3.42 Å from the first graphitic layer, in agreement with previous studies.^130^ Notably, during the adsorption process, we observe the intramolecular hydrogen transfer between the γ-carboxyl group (green circle in Figure 1b) and the N8 atom (blue circle in Figure 1b).

**Figure 9:**
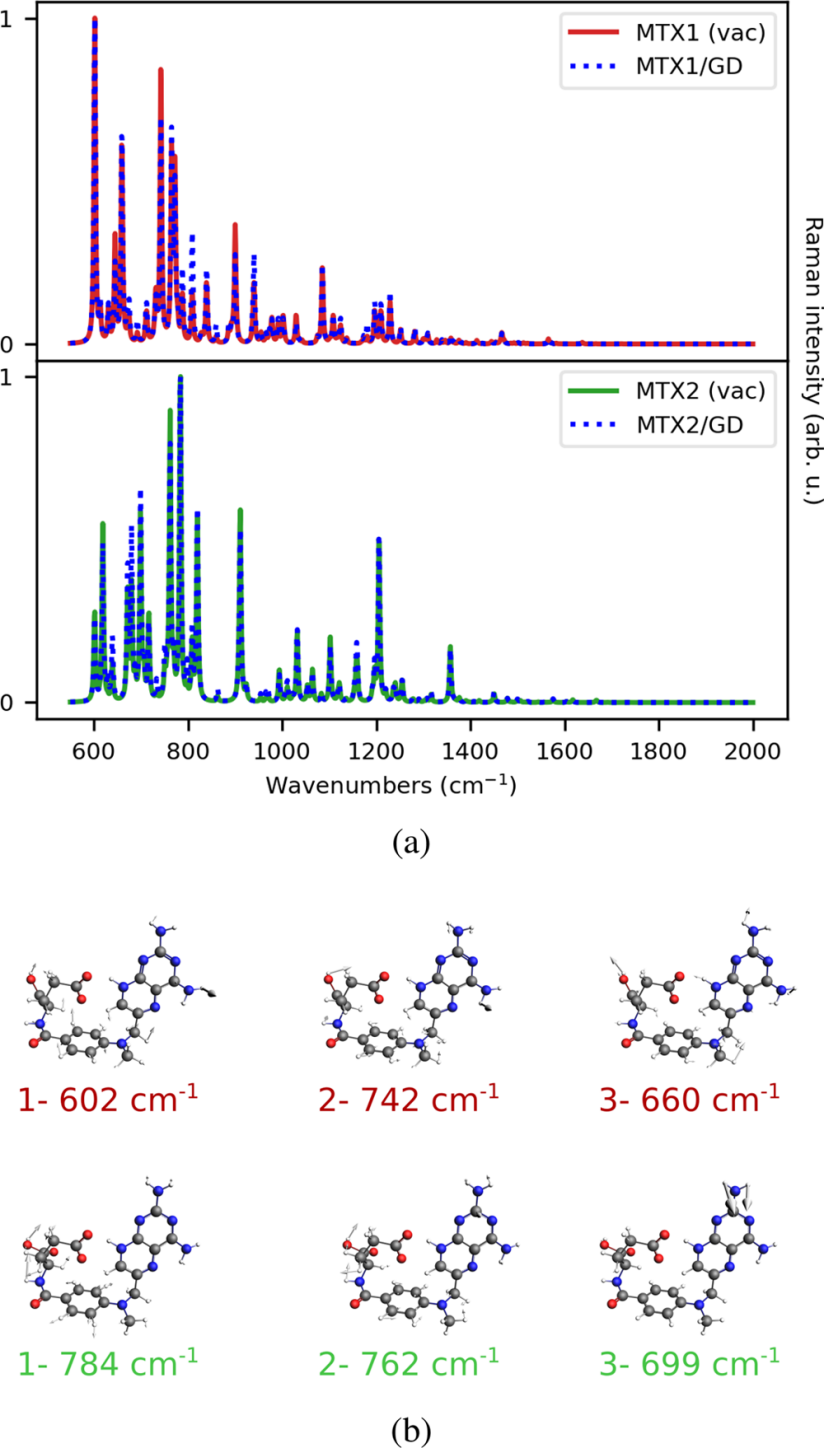
(a) Normalized MTX1 and MTX2 Raman spectra as computed in vacuo (solid line) and adsorbed on GD16 (dashed line). (b) MTX1 and MTX2 normal modes associated with the highest Raman intensities. The Raman signal is computed at GD18 PRF (0.24 eV, see also Table S3 in the SI).

After MTX adsorption, we extract 5000 snapshots from the remaining 250 ps of the production run. MTX geometries are processed by means of the GROMOS^131^ clustering approach as it is implemented in the 2020.3 version of the GROMACS^132,133^ software. As a result, only two MTX geometries are selected, representing 68.7% (MTX1) and 28.8% (MTX2) of the total configurational space. The two structures are reported in Figure 9b (see also Figure S9 in the SI).

To simulate MTX GERS, we remove the graphitic substrate, and we substitute the first layer by a perfect GD with *r* = 16 nm (GD16, see Table S3 in the SI) on the center of which MTX is adsorbed. MTX1 and MTX2 Raman (in gas phase) and GERS spectra are finally computed at the B3LYP/DZP level of theory, by setting *E*_*F*_ = 0.4 eV (PRF = 0.24 eV). Spectra are reported in Figure 9a.

**Figure 10:**
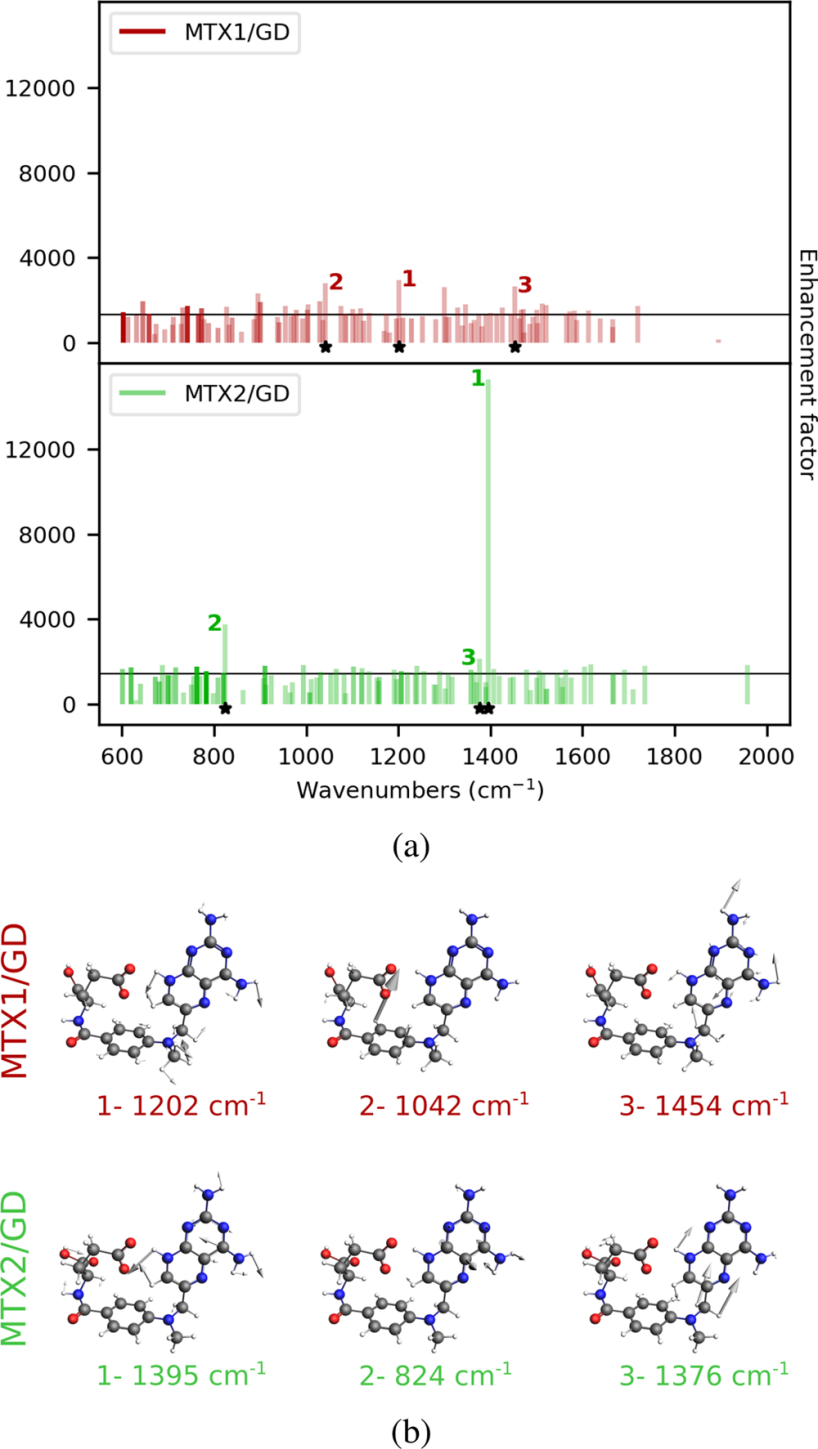
(a) Computed QM/ωFQ MTX1/GD18 (top) and MTX2/GD18 (bottom) normal modes’ EFs and AEF reported as horizontal black lines. The darkness of each bar is proportional to the Raman intensity. (b) MTX1 and MTX2 normal modes associated with the largest EFs.

MTX1 and MTX2 Raman spectra, both in the gas phase and adsorbed on GD16, qualitatively differ, as they are characterized by a shift in vibrational frequencies, and in relative intensities of the main bands. Indeed, while the MTX1 Raman spectrum is dominated by the presence of intense bands in the low energy region (between 600 and 800 cm^–1^), the MTX2 spectrum reports diverse intense peaks in the whole considered spectral range. The different MTX1 and MTX2 spectral fingerprints reflect the intrinsic dissimilarity in MTX conformations, as expected from the clustering process. When moving from the gas phase to the adsorbed configuration, both MTX1 and MTX2 Raman spectra are enhanced by an AEF of about 900. However, Raman spectra undergo small qualitative variations. Such findings can be rationalized by considering that the plasmonic electric field generated by the GD is strongly inhomogeneous at the GD edges, while at the GD center it is almost uniform. Therefore, we expect that the electric field felt by each normal mode is essentially uniform, although more intense than in vacuo. To analyze this hypothesis from a quantitative point of view, we compute for both structures the EFs associated with each normal mode (see eq 28), which are graphically depicted in Figure 10a. In particular, for each normal mode, EFs are plotted with a color scale that follows the intensities of the corresponding Raman peak.

Except for a few outliers, all computed EFs are almost equal independent of the normal mode and fall around the AEF (about 900). The normal modes featuring the highest EF are depicted in Figure 10b. However, these modes are associated with rather low-intensity bands. On the contrary, EFs computed for the most intense Raman peaks, which dominate the computed spectra, are almost constant at a value of around 900. As it can be noticed, it is difficult to find a correlation between the most intense normal modes of both conformers because they are localized on separated regions of the MTX structure, independent of the distance with respect to the GD surface. In light of the above discussion, this is not surprising. In fact, the presence of the GD yields an almost uniform increase of the Raman bands as compared to the case in vacuo, without greatly affecting their relative intensities. Thus, the most intense peaks are related to the same normal modes that provide the most intense bands in the gas-phase Raman spectrum.

**Figure 11:**
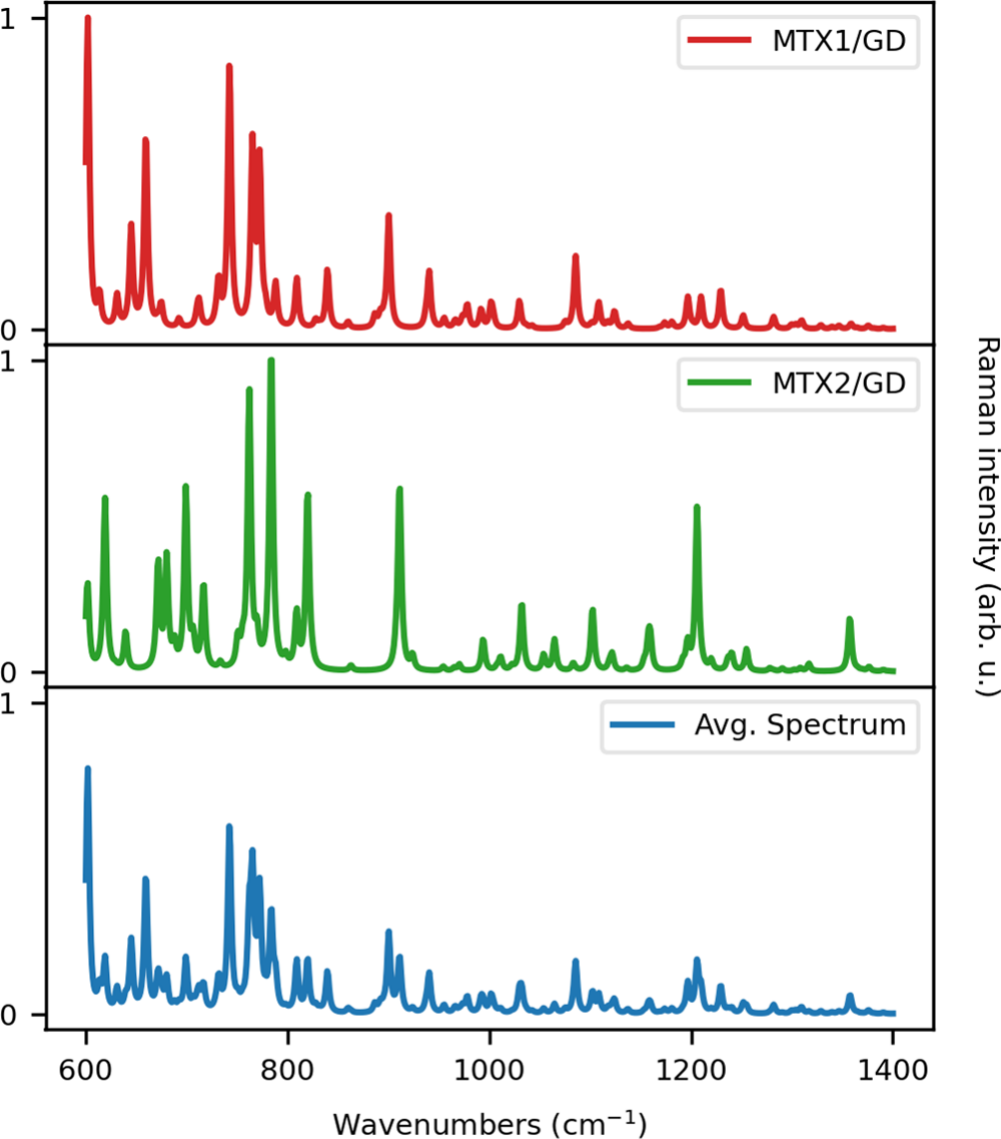
Normalized QM/ωFQFμ GERS spectra of MTX1 and MTX2. The weighted average spectrum is also displayed (bottom panel). The GERS signal is computed at GD18 PRF (0.24 eV, see also Table S3 in the SI).

To conclude the discussion, in Figure 11 we report the MTX/GD16 GERS spectrum as obtained by averaging MTX1 and MTX2 spectra, which are also graphically depicted.

The most important contribution to the Raman spectrum is due to the MTX1 structure (68.7%). Therefore, only the most intense peaks of MTX2 arise noticeably in the averaged spectrum. MTX2 bands, however, drastically affect the final GERS spectrum, which does not present anymore the spectral fingerprints related only to MTX1 or MTX2. Moreover, the final spectrum is particularly noisy, especially in the high-frequency region. This is essentially due to small differences between the vibrational frequencies of the two conformers.

Finally, it is worth pointing out that, for the system under consideration, graphene is able to provide a significant EM enhancement, comparable to specific metal NPs, such as Au surfaces.^134^

## 5 Summary, Conclusions, and Future Perspectives

In this work, we have presented a new methodology to simulate the Raman spectrum of molecular systems, described at the QM level, adsorbed on plasmonic substrates, treated in terms of ωFQ and ωFQFμ classical atomistic and frequency-dependent force fields. The resulting multiscale approach is particularly versatile because the underlying ωFQ(Fμ) approach is able to accurately describe the plasmonic response of both metallic nanostructures and 2D graphene-based substrates.^63−65,68^ The performance of QM/ωFQ(Fμ) has been tested on PY adsorbed on Ag/Au nanostructures and graphene disks. The enhancement factors of the Raman intensities have been studied as a function of the PS size, the PY-PS distance, and the relative PY-PS orientation, and the qualitative trend has been compared with the *E*^4^ approximation. The largest enhancement has been obtained for silver cTO structures, with an average enhancement factor of about 5000. Such an analysis has allowed confirming that the developed approach is able to correctly describe the EM enhancement in SERS/GERS spectroscopies. Thus, as a final application, we have presented an application of the approach to a realistic case, i.e. methotrexate adsorbed on a graphene disk, for which we also investigate the configurational populations as computed by means of ReaxFF MD simulations. The computed GERS spectrum shows that, although GD is able to enhance the Raman bands by a non-negligible factor of about 900, the GD substrate is not able to discriminate between vibrational normal modes.

Our approach has the potential to become a reliable and efficient tool for the simulation of optical properties of molecular systems adsorbed on plasmonic substrates and might become a viable tool for the experimental design of innovative nanostructured materials able to maximize the enhancement of Raman intensities of a target molecule. To this end, it is worth noting that the current computational bottleneck in QM/ωFQFμ is the cost associated with the solution of ωFQ/ωFQFμ linear systems (see eqs 1 and 9), which can be overcome by resorting to efficient approaches that have been recently tested by some of the authors.^68^ In particular, with the aid of on-the-fly iterative techniques, we can largely increase the size of the treatable plasmonic nanostructures, to reach the typical dimensions exploited in real experiments. The latter would also benefit from taking into account the interaction of the molecular system and substrate with a solvating environment.

## Supporting Information Available

List of abbreviations introduced in the main text; geometrical details and PRFs on the PS presented in the main text; robustness of the computed EFs with respect to the numerical differentiation step size; Raman spectra of PY-NP composite system with different levels of theory; numerical consistency of the computed SERS spectra and AEF with respect to the DFT integration grid; graphical representation of the isolated PY normal modes computed at the B3LYP/DZP level of theory; EFs for selected PY-PS configurations; absolute AEF and Υ_vol_^4^ values for PY adsorbed on Ag NPs and GDs; correlation of AEF and *E*^4^ approximation for Au NPs with respect to the size of the PS; absolute AEF and Υ_vol_^4^ values for PY adsorbed on Ag_10179_ in the Ih morphology; correlation of AEF and *E*^4^ approximation for the NPs with respect to the PY-Au distance; AEF and SERS spectra of PY adsorbed on Ag_10179_ in the Ih morphology by changing the relative position of the two moieties; comparison of MTX1 and MTX2 structures; comment on the range of validity of the *E*^4^ approximation with respect to the AEF estimate (PDF)

## References

[ref1] Fleischmann M.; Hendra P. J.; McQuillan A. J. Raman spectra of pyridine adsorbed at a silver electrode. Chem. Phys. Lett. 1974, 26, 163–166. 10.1016/0009-2614(74)85388-1.

[ref2] Le Ru E. C.; Blackie E.; Meyer M.; Etchegoin P. G. Surface enhanced Raman scattering enhancement factors: a comprehensive study. J. Phys. Chem. C 2007, 111, 13794–13803. 10.1021/jp0687908.

[ref3] LeRu E.; Etchegoin P. Principles of Surface Enhanced Raman Spectroscopy; Elsevier, 2009.

[ref4] Ding S.-Y.; You E.-M.; Tian Z.-Q.; Moskovits M. Electromagnetic theories of surface-enhanced Raman spectroscopy. Chem. Soc. Rev. 2017, 46, 4042–4076. 10.1039/C7CS00238F. 28660954

[ref5] Zrimsek A. B.; Chiang N.; Mattei M.; Zaleski S.; McAnally M. O.; Chapman C. T.; Henry A.-I.; Schatz G. C.; Van Duyne R. P. Single-molecule chemistry with surface-and tip-enhanced Raman spectroscopy. Chem. Rev. 2017, 117, 7583–7613. 10.1021/acs.chemrev.6b00552. 28610424

[ref6] Wang X.; Shi W.; She G.; Mu L.; Lee S. High-performance surface-enhanced Raman scattering sensors based on Ag nanoparticles-coated Si nanowire arrays for quantitative detection of pesticides. Appl. Phys. Lett. 2010, 96, 053104 10.1063/1.3300837.

[ref7] Wang Y.; Wang Y.; Wang W.; Sun K.; Chen L. Reporter-embedded SERS tags from gold nanorod seeds: selective immobilization of reporter molecules at the tip of nanorods. ACS Appl. Mater. 2016, 8, 28105–28115. 10.1021/acsami.6b04216. 27696805

[ref8] Zhang X.; Young M. A.; Lyandres O.; Van Duyne R. P. Rapid detection of an anthrax biomarker by surface-enhanced Raman spectroscopy. J. Am. Chem. Soc. 2005, 127, 4484–4489. 10.1021/ja043623b. 15783231

[ref9] Jarvis R. M.; Goodacre R. Discrimination of bacteria using surface-enhanced Raman spectroscopy. Anal. Chem. 2004, 76, 40–47. 10.1021/ac034689c. 14697030

[ref10] Huh Y. S.; Chung A. J.; Erickson D. Surface enhanced Raman spectroscopy and its application to molecular and cellular analysis. Microfluid Nanofluidic 2009, 6, 285–297. 10.1007/s10404-008-0392-3.

[ref11] Cailletaud J.; De Bleye C.; Dumont E.; Sacré P.-Y.; Netchacovitch L.; Gut Y.; Boiret M.; Ginot Y.-M.; Hubert P.; Ziemons E. Critical review of surface-enhanced Raman spectroscopy applications in the pharmaceutical field. J. Pharm. Biomed. Anal. 2018, 147, 458–472. 10.1016/j.jpba.2017.06.056. 28688617

[ref12] Han X. X.; Rodriguez R. S.; Haynes C. L.; Ozaki Y.; Zhao B. Surface-enhanced Raman spectroscopy. Nat. Rev. Methods Primers 2021, 1, 1–17. 10.1038/s43586-021-00083-6.

[ref13] Sharma B.; Frontiera R. R.; Henry A.-I.; Ringe E.; Van Duyne R. P. SERS: Materials, applications, and the future. Mater. Today 2012, 15, 16–25. 10.1016/S1369-7021(12)70017-2.

[ref14] Le Ru E. C.; Etchegoin P. G. Single-molecule surface-enhanced Raman spectroscopy. Annu. Rev. Phys. Chem. 2012, 63, 65–87. 10.1146/annurev-physchem-032511-143757. 22224704

[ref15] Langer J.; Jimenez deAberasturi D.; Aizpurua J.; Alvarez-Puebla R. A.; Auguié B.; Baumberg J. J.; Bazan G. C.; Bell S. E.; Boisen A.; Brolo A. G.; et al. Present and future of surface-enhanced Raman scattering. ACS Nano 2020, 14, 28–117. 10.1021/acsnano.9b04224. 31478375 PMC6990571

[ref16] Pilot R.; Signorini R.; Durante C.; Orian L.; Bhamidipati M.; Fabris L. A review on surface-enhanced Raman scattering. Biosensors 2019, 9, 57 10.3390/bios9020057. 30999661 PMC6627380

[ref17] Zong C.; Xu M.; Xu L.-J.; Wei T.; Ma X.; Zheng X.-S.; Hu R.; Ren B. Surface-enhanced Raman spectroscopy for bioanalysis: reliability and challenges. Chem. Rev. 2018, 118, 4946–4980. 10.1021/acs.chemrev.7b00668. 29638112

[ref18] Albrecht M. G.; Creighton J. A. Anomalously intense Raman spectra of pyridine at a silver electrode. J. Am. Chem. Soc. 1977, 99, 5215–5217. 10.1021/ja00457a071.

[ref19] Jeanmaire D. L.; Van Duyne R. P. Surface Raman spectroelectrochemistry: Part I. Heterocyclic, aromatic, and aliphatic amines adsorbed on the anodized silver electrode. J. Electroanal. Chem. Interface Electrochem. 1977, 84, 1–20. 10.1016/S0022-0728(77)80224-6.

[ref20] Campion A.; Kambhampati P. Surface-enhanced Raman scattering. Chem. Soc. Rev. 1998, 27, 241–250. 10.1039/a827241z.

[ref21] Willets K. A.; Van Duyne R. P. Localized surface plasmon resonance spectroscopy and sensing. Annu. Rev. Phys. Chem. 2007, 58, 267–297. 10.1146/annurev.physchem.58.032806.104607. 17067281

[ref22] Alvarez-Puebla R.; Liz-Marzán L. M.; García de Abajo F. J. Light concentration at the nanometer scale. J. Phys. Chem. Lett. 2010, 1, 2428–2434. 10.1021/jz100820m.

[ref23] Le Ru E.; Etchegoin P. Principles of Surface-Enhanced Raman Spectroscopy: and related plasmonic effects; Elsevier, 2008.

[ref24] Maier S. A. Plasmonics: fundamentals and applications; Springer Science & Business Media, 2007.

[ref25] Junno T.; Deppert K.; Montelius L.; Samuelson L. Controlled manipulation of nanoparticles with an atomic force microscope. Appl. Phys. Lett. 1995, 66, 3627–3629. 10.1063/1.113809.

[ref26] Ishida T.; Murayama T.; Taketoshi A.; Haruta M. Importance of size and contact structure of gold nanoparticles for the genesis of unique catalytic processes. Chem. Rev. 2020, 120, 464–525. 10.1021/acs.chemrev.9b00551. 31820953

[ref27] Sau T. K.; Rogach A. L. Nonspherical noble metal nanoparticles: colloid-chemical synthesis and morphology control. Adv. Mater. 2010, 22, 1781–1804. 10.1002/adma.200901271. 20512953

[ref28] Liz-Marzán L. M. Tailoring surface plasmons through the morphology and assembly of metal nanoparticles. Langmuir 2006, 22, 32–41. 10.1021/la0513353. 16378396

[ref29] Grzelczak M.; Pérez-Juste J.; Mulvaney P.; Liz-Marzán L. M. Shape control in gold nanoparticle synthesis. Chem. Soc. Rev. 2008, 37, 1783–1791. 10.1039/b711490g. 18762828

[ref30] Blackie E. J.; Le Ru E. C.; Etchegoin P. G. Single-molecule surface-enhanced Raman spectroscopy of nonresonant molecules. J. Am. Chem. Soc. 2009, 131, 14466–14472. 10.1021/ja905319w. 19807188

[ref31] Fesenko O.; Dovbeshko G.; Dementjev A.; Karpicz R.; Kaplas T.; Svirko Y. Graphene-enhanced Raman spectroscopy of thymine adsorbed on single-layer graphene. Nanoscale Res. Lett. 2015, 10, 1–7. 10.1186/s11671-015-0869-4. 25897307 PMC4398685

[ref32] Lai H.; Xu F.; Zhang Y.; Wang L. Recent progress on graphene-based substrates for surface-enhanced Raman scattering applications. J. Mater. Chem. B 2018, 6, 4008–4028. 10.1039/C8TB00902C. 32255147

[ref33] Chen N.; Xiao T.-H.; Luo Z.; Kitahama Y.; Hiramatsu K.; Kishimoto N.; Itoh T.; Cheng Z.; Goda K. Porous carbon nanowire array for surface-enhanced Raman spectroscopy. Nat. Commun. 2020, 11, 1–8. 10.1038/s41467-020-18590-7. 32973145 PMC7519110

[ref34] Payton J. L.; Morton S. M.; Moore J. E.; Jensen L. A discrete interaction model/quantum mechanical method for simulating surface-enhanced Raman spectroscopy. J. Chem. Phys. 2012, 136, 214103 10.1063/1.4722755. 22697526

[ref35] Payton J. L.; Morton S. M.; Moore J. E.; Jensen L. A hybrid atomistic electrodynamics–quantum mechanical approach for simulating surface-enhanced Raman scattering. Acc. Chem. Res. 2014, 47, 88–99. 10.1021/ar400075r. 23965411

[ref36] Hu Z.; Chulhai D. V.; Jensen L. Simulating surface-enhanced hyper-Raman scattering using atomistic electrodynamics-quantum mechanical models. J. Chem. Theory Comput. 2016, 12, 5968–5978. 10.1021/acs.jctc.6b00940. 27792337

[ref37] Corni S.; Tomasi J. Enhanced response properties of a chromophore physisorbed on a metal particle. J. Chem. Phys. 2001, 114, 3739–3751. 10.1063/1.1342241.

[ref38] Corni S.; Tomasi J. Surface enhanced Raman scattering from a single molecule adsorbed on a metal particle aggregate: A theoretical study. J. Chem. Phys. 2002, 116, 1156–1164. 10.1063/1.1428349.

[ref39] Corni S.; Tomasi J. Excitation energies of a molecule close to a metal surface. J. Chem. Phys. 2002, 117, 7266–7278. 10.1063/1.1507579.

[ref40] Corni S.; Tomasi J. Theoretical evaluation of Raman spectra and enhancement factors for a molecule adsorbed on a complex-shaped metal particle. Chem. Phys. Lett. 2001, 342, 135–140. 10.1016/S0009-2614(01)00582-6.

[ref41] Corni S.; Tomasi J. Erratum to: Theoretical evaluation of Raman spectra and enhancement factors for a molecule adsorbed on a complex-shaped metal particle [Chem. Phys. Lett. 342 (2001) 135–140]. Chem. Phys. Lett. 2002, 365, 552–553. 10.1016/S0009-2614(02)01506-3.

[ref42] Chen R.; Jensen L. Interpreting the chemical mechanism in SERS using a Raman bond model. J. Chem. Phys. 2020, 152, 024126 10.1063/1.5138204. 31941295

[ref43] Chen R.; Jensen L. Understanding chemical enhancements of surface-enhanced Raman scattering using a Raman bond model for extended systems. J. Chem. Phys. 2022, 157, 184705 10.1063/5.0124553. 36379771

[ref44] Becca J. C.; Chen X.; Jensen L. A discrete interaction model/quantum mechanical method for simulating surface-enhanced Raman spectroscopy in solution. J. Chem. Phys. 2021, 154, 224705 10.1063/5.0051256. 34241237

[ref45] Liu P.; Chulhai D. V.; Jensen L. Single-molecule imaging using atomistic near-field tip-enhanced Raman spectroscopy. ACS Nano 2017, 11, 5094–5102. 10.1021/acsnano.7b02058. 28463555

[ref46] Chulhai D. V.; Chen X.; Jensen L. Simulating ensemble-averaged surface-enhanced Raman scattering. J. Phys. Chem. C 2016, 120, 20833–20842. 10.1021/acs.jpcc.6b02159.

[ref47] Zhao L.; Jensen L.; Schatz G. C. Pyridine- Ag20 cluster: a model system for studying surface-enhanced Raman scattering. J. Am. Chem. Soc. 2006, 128, 2911–2919. 10.1021/ja0556326. 16506770

[ref48] Jensen L.; Aikens C. M.; Schatz G. C. Electronic structure methods for studying surface-enhanced Raman scattering. Chem. Soc. Rev. 2008, 37, 1061–1073. 10.1039/b706023h. 18443690

[ref49] Morton S. M.; Jensen L. Understanding the molecule- surface chemical coupling in SERS. J. Am. Chem. Soc. 2009, 131, 4090–4098. 10.1021/ja809143c. 19254020

[ref50] Morton S. M.; Silverstein D. W.; Jensen L. Theoretical studies of plasmonics using electronic structure methods. Chem. Rev. 2011, 111, 3962–3994. 10.1021/cr100265f. 21344862

[ref51] Jørgensen S.; Ratner M. A.; Mikkelsen K. V. Heterogeneous solvation: An ab initio approach. J. Chem. Phys. 2001, 115, 3792–3803. 10.1063/1.1387979.

[ref52] Neuhauser D.; Lopata K. Molecular nanopolaritonics: Cross manipulation of near-field plasmons and molecules. I. Theory and application to junction control. J. Chem. Phys. 2007, 127, 154715 10.1063/1.2790436. 17949199

[ref53] Masiello D. J.; Schatz G. C. Many-body theory of surface-enhanced Raman scattering. Phys. Rev. A 2008, 78, 042505 10.1103/PhysRevA.78.042505.

[ref54] Vukovic S.; Corni S.; Mennucci B. Fluorescence enhancement of chromophores close to metal nanoparticles. Optimal setup revealed by the polarizable continuum model. J. Phys. Chem. C 2009, 113, 121–133. 10.1021/jp808116y.

[ref55] Lopata K.; Neuhauser D. Multiscale Maxwell–Schrödinger modeling: A split field finite-difference time-domain approach to molecular nanopolaritonics. J. Chem. Phys. 2009, 130, 104707 10.1063/1.3082245. 19292549

[ref56] Arcisauskaite V.; Kongsted J.; Hansen T.; Mikkelsen K. V. Charge transfer excitation energies in pyridine–silver complexes studied by a QM/MM method. Chem. Phys. Lett. 2009, 470, 285–288. 10.1016/j.cplett.2009.01.067.

[ref57] Masiello D. J.; Schatz G. C. On the linear response and scattering of an interacting molecule-metal system. J. Chem. Phys. 2010, 132, 064102 10.1063/1.3308624. 20151728

[ref58] Chen H.; McMahon J. M.; Ratner M. A.; Schatz G. C. Classical electrodynamics coupled to quantum mechanics for calculation of molecular optical properties: a RT-TDDFT/FDTD approach. J. Phys. Chem. C 2010, 114, 14384–14392. 10.1021/jp1043392.

[ref59] Hao Q.; Morton S. M.; Wang B.; Zhao Y.; Jensen L.; Jun Huang T. Tuning surface-enhanced Raman scattering from graphene substrates using the electric field effect and chemical doping. Appl. Phys. Lett. 2013, 102, 011102 10.1063/1.4755756. 23382597 PMC3548806

[ref60] Bonatti L.; Nicoli L.; Giovannini T.; Cappelli C. In silico design of graphene plasmonic hot-spots. Nanoscale Adv. 2022, 4, 2294–2302. 10.1039/D2NA00088A. 35706845 PMC9113057

[ref61] Giovannini T.; Bonatti L.; Lafiosca P.; Nicoli L.; Castagnola M.; Illobre P. G.; Corni S.; Cappelli C. Do We Really Need Quantum Mechanics to Describe Plasmonic Properties of Metal Nanostructures?. ACS Photonics 2022, 9, 3025 10.1021/acsphotonics.2c00761. 36164484 PMC9502030

[ref62] Lombardi J. R.; Birke R. L.; Lu T.; Xu J. Charge-transfer theory of surface enhanced Raman spectroscopy: Herzberg–Teller contributions. J. Chem. Phys. 1986, 84, 4174–4180. 10.1063/1.450037.

[ref63] Giovannini T.; Rosa M.; Corni S.; Cappelli C. A classical picture of subnanometer junctions: an atomistic Drude approach to nanoplasmonics. Nanoscale 2019, 11, 6004–6015. 10.1039/C8NR09134J. 30869089

[ref64] Giovannini T.; Bonatti L.; Polini M.; Cappelli C. Graphene plasmonics: Fully atomistic approach for realistic structures. J. Phys. Chem. Lett. 2020, 11, 7595–7602. 10.1021/acs.jpclett.0c02051. 32805117 PMC7503861

[ref65] Bonatti L.; Gil G.; Giovannini T.; Corni S.; Cappelli C. Plasmonic resonances of metal nanoparticles: atomistic vs. Continuum approaches. Front. Chem. 2020, 8, 340 10.3389/fchem.2020.00340. 32457870 PMC7221199

[ref66] Zanotto S.; Bonatti L.; Pantano M. F.; Miseikis V.; Speranza G.; Giovannini T.; Coletti C.; Cappelli C.; Tredicucci A.; Toncelli A. Strain-Induced Plasmon Confinement in Polycrystalline Graphene. ACS Photonics 2023, 10, 394 10.1021/acsphotonics.2c01157. 36820323 PMC9936574

[ref67] Barros E. B.; Dresselhaus M. S. Theory of Raman enhancement by two-dimensional materials: Applications for graphene-enhanced Raman spectroscopy. Phys. Rev. B 2014, 90, 035443 10.1103/PhysRevB.90.035443.

[ref68] Lafiosca P.; Giovannini T.; Benzi M.; Cappelli C. Going Beyond the Limits of Classical Atomistic Modeling of Plasmonic Nanostructures. J. Phys. Chem. C 2021, 125, 23848 10.1021/acs.jpcc.1c04716. PMC8573767 34765073

[ref69] Jackson J. D. Classical electrodynamics; John Wiley & Sons, 1999.

[ref70] Giovannini T.; Puglisi A.; Ambrosetti M.; Cappelli C. Polarizable QM/MM approach with fluctuating charges and fluctuating dipoles: the QM/FQFμ model. J. Chem. Theory Comput. 2019, 15, 2233–2245. 10.1021/acs.jctc.8b01149. 30875213

[ref71] Pelton M.; Bryant G. W. Introduction to metal-nanoparticle plasmonics; John Wiley & Sons, 2013; Vol. 5.

[ref72] Castro Neto A. H.; Guinea F.; Peres N. M. R.; Novoselov K. S.; Geim A. K. The electronic properties of graphene. Rev. Mod. Phys. 2009, 81, 109 10.1103/RevModPhys.81.109.

[ref73] Pinchuk A.; Kreibig U.; Hilger A. Optical properties of metallic nanoparticles: influence of interface effects and interband transitions. Surf. Sci. 2004, 557, 269–280. 10.1016/j.susc.2004.03.056.

[ref74] Pinchuk A.; Von Plessen G.; Kreibig U. Influence of interband electronic transitions on the optical absorption in metallic nanoparticles. J. Phys. D: Appl. Phys. 2004, 37, 3133 10.1088/0022-3727/37/22/012.

[ref75] Balamurugan B.; Maruyama T. Evidence of an enhanced interband absorption in Au nanoparticles: size-dependent electronic structure and optical properties. Appl. Phys. Lett. 2005, 87, 143105 10.1063/1.2077834.

[ref76] Liebsch A. Surface-plasmon dispersion and size dependence of Mie resonance: silver versus simple metals. Phys. Rev. B 1993, 48, 11317 10.1103/PhysRevB.48.11317. 10007444

[ref77] Santiago E. Y.; Besteiro L. V.; Kong X.-T.; Correa-Duarte M. A.; Wang Z.; Govorov A. O. Efficiency of hot-electron generation in plasmonic nanocrystals with complex shapes: surface-induced scattering, hot spots, and interband transitions. ACS Photonics 2020, 7, 2807–2824. 10.1021/acsphotonics.0c01065.

[ref78] Warshel A.; Levitt M. Theoretical studies of enzymic reactions: dielectric, electrostatic and steric stabilization of the carbonium ion in the reaction of lysozyme. J. Mol. Biol. 1976, 103, 227–249. 10.1016/0022-2836(76)90311-9. 985660

[ref79] Lin H.; Truhlar D. G. QM/MM: what have we learned, where are we, and where do we go from here?. Theor. Chem. Acc. 2007, 117, 185–199. 10.1007/s00214-006-0143-z.

[ref80] Senn H. M.; Thiel W. QM/MM methods for biomolecular systems. Angew. Chem., Int. Ed. 2009, 48, 1198–1229. 10.1002/anie.200802019. 19173328

[ref81] Mennucci B.; Corni S. Multiscale modelling of photoinduced processes in composite systems. Nat. Rev. Chem. 2019, 3, 315–330. 10.1038/s41570-019-0092-4.

[ref82] Morton S. M.; Jensen L. A discrete interaction model/quantum mechanical method for describing response properties of molecules adsorbed on metal nanoparticles. J. Chem. Phys. 2010, 133, 074103 10.1063/1.3457365. 20726631

[ref83] Guido C. A.; Rosa M.; Cammi R.; Corni S. An open quantum system theory for polarizable continuum models. J. Chem. Phys. 2020, 152, 174114 10.1063/5.0003523. 32384839

[ref84] Coccia E.; Fregoni J.; Guido C.; Marsili M.; Pipolo S.; Corni S. Hybrid theoretical models for molecular nanoplasmonics. J. Chem. Phys. 2020, 153, 200901 10.1063/5.0027935. 33261492

[ref85] Corni S.; Pipolo S.; Cammi R. Equation of motion for the solvent polarization apparent charges in the polarizable continuum model: Application to real-time TDDFT. J. Phys. Chem. A 2015, 119, 5405–5416. 10.1021/jp5106828. 25485456

[ref86] Rick S. W.; Stuart S. J.; Bader J. S.; Berne B. Fluctuating charge force fields for aqueous solutions. J. Mol. Liq. 1995, 65, 31–40. 10.1016/0167-7322(95)00842-7.

[ref87] Cappelli C. Integrated QM/polarizable MM/continuum approaches to model chiroptical properties of strongly interacting solute–solvent systems. Int. J. Quantum Chem. 2016, 116, 1532–1542. 10.1002/qua.25199.

[ref88] Giovannini T.; Egidi F.; Cappelli C. Molecular spectroscopy of aqueous solutions: a theoretical perspective. Chem. Soc. Rev. 2020, 49, 5664–5677. 10.1039/C9CS00464E. 32744278

[ref89] Giovannini T.; Egidi F.; Cappelli C. Theory and algorithms for chiroptical properties and spectroscopies of aqueous systems. Phys. Chem. Chem. Phys. 2020, 22, 22864–22879. 10.1039/D0CP04027D. 33043930

[ref90] Giovannini T.; Riso R. R.; Ambrosetti M.; Puglisi A.; Cappelli C. Electronic transitions for a fully polarizable qm/mm approach based on fluctuating charges and fluctuating dipoles: linear and corrected linear response regimes. J. Chem. Phys. 2019, 151, 174104 10.1063/1.5121396. 31703497

[ref91] Giovannini T.; Grazioli L.; Ambrosetti M.; Cappelli C. Calculation of ir spectra with a fully polarizable qm/mm approach based on fluctuating charges and fluctuating dipoles. J. Chem. Theory Comput. 2019, 15, 5495–5507. 10.1021/acs.jctc.9b00574. 31436976

[ref92] Giovannini T.; Ambrosetti M.; Cappelli C. Quantum confinement effects on solvatochromic shifts of molecular solutes. J. Phys. Chem. Lett. 2019, 10, 5823–5829. 10.1021/acs.jpclett.9b02318. 31518133

[ref93] Marrazzini G.; Giovannini T.; Egidi F.; Cappelli C. Calculation of linear and non-linear electric response properties of systems in aqueous solution: A polarizable quantum/classical approach with quantum repulsion effects. J. Chem. Theory Comput. 2020, 16, 6993–7004. 10.1021/acs.jctc.0c00674. 33058671 PMC8015238

[ref94] te Velde G.; Bickelhaupt F. M.; Baerends E. J.; Fonseca Guerra C.; van Gisbergen S. J. A.; Snijders J. G.; Ziegler T. Chemistry with ADF. J. Comput. Chem. 2001, 22, 931–967. 10.1002/jcc.1056.

[ref95] Baerends E.; ADF (version 2020.x); Theoretical Chemistry; Vrije Universiteit: Amsterdam, The Netherlands, 2020. http://www.scm.com.

[ref96] Nicoli L.; Giovannini T.; Cappelli C. Assessing the Quality of QM/MM Approaches to Describe Vacuo-to-water Solvatochromic Shifts. J. Chem. Phys. 2022, 157, 214101 10.1063/5.0118664. 36511555

[ref97] Jensen L.; Van Duijnen P. T.; Snijders J. G. A discrete solvent reaction field model within density functional theory. J. Chem. Phys. 2003, 118, 514–521. 10.1063/1.1527010.

[ref98] Casida M. E. Recent Advances In Density Functional Methods: (Part I); World Scientific, 1995; pp 155–192.

[ref99] Norman P.; Ruud K.; Saue T. Principles and practices of molecular properties: Theory, modeling, and simulations; John Wiley & Sons, 2018.

[ref100] Giovannini T.; Ambrosetti M.; Cappelli C. A polarizable embedding approach to second harmonic generation (SHG) of molecular systems in aqueous solutions. Theor. Chem. Acc. 2018, 137, 1–11. 10.1007/s00214-018-2247-7.

[ref101] Morton S. M.; Jensen L. A discrete interaction model/quantum mechanical method to describe the interaction of metal nanoparticles and molecular absorption. J. Chem. Phys. 2011, 135, 134103 10.1063/1.3643381. 21992278

[ref102] Placzek G.; Teller E. Die Rotationsstruktur der Ramanbanden mehratomiger Moleküle. Zeitschrift für Physik 1933, 81, 209–258. 10.1007/BF01338366.

[ref103] Placzek G. In Handbuch der Radiologie; Marx G., Ed.; Akademische Verlagsgesellschaft: Leipzig, 1934.

[ref104] Jensen L.; Zhao L.; Autschbach J.; Schatz G. Theory and method for calculating resonance Raman scattering from resonance polarizability derivatives. J. Chem. Phys. 2005, 123, 174110 10.1063/1.2046670. 16375520

[ref105] Corni S.; Tomasi J. Surface-Enhanced Raman Scattering: Physics and Applications; Springer, 2006; pp 105–123.

[ref106] Louden P.; Bhattarai H.; Neidhart S.; Lin T.; Vardeman C. F. II; Fennell C. J.; Meineke M. A.; Kuang S.; Lamichhane M.; Michalka J.; OPENMD-2.5: molecular dynamics in the open; OpenMD, 2017. http://openmd.org.

[ref107] Humphrey W.; Dalke A.; Schulten K. VMD – Visual Molecular Dynamics. J. Mol. Graphics 1996, 14, 33–38. 10.1016/0263-7855(96)00018-5. 8744570

[ref108] Van Lenthe E.; Baerends E. J. Optimized Slater-type basis sets for the elements 1–118. J. Comput. Chem. 2003, 24, 1142–1156. 10.1002/jcc.10255. 12759913

[ref109] Van Gisbergen S.; Snijders J.; Baerends E. A density functional theory study of frequency-dependent polarizabilities and Van der Waals dispersion coefficients for polyatomic molecules. J. Chem. Phys. 1995, 103, 9347–9354. 10.1063/1.469994.

[ref110] Van Gisbergen S.; Snijders J.; Baerends E. Implementation of time-dependent density functional response equations. Comput. Phys. Commun. 1999, 118, 119–138. 10.1016/S0010-4655(99)00187-3.

[ref111] Fan L.; Ziegler T. Application of density functional theory to infrared absorption intensity calculations on main group molecules. J. Chem. Phys. 1992, 96, 9005–9012. 10.1063/1.462258.

[ref112] Fan L.; Ziegler T. Application of density functional theory to infrared absorption intensity calculations on transition-metal carbonyls. J. Phys. Chem. 1992, 96, 6937–6941. 10.1021/j100196a016.

[ref113] Van Gisbergen S.; Snijders J.; Baerends E. Application of time-dependent density functional response theory to Raman scattering. Chem. Phys. Lett. 1996, 259, 599–604. 10.1016/0009-2614(96)00858-5.

[ref114] Van Duyne R.; Jeanmaire D. Surface Raman spectroelectochemistry: part1. heterocyclic, aromatic, and aliphatic Amines adsorbed on the anodized silver electrode. J. Electroanal. Chem. 1977, 84, 1–20. 10.1016/S0022-0728(77)80224-6.

[ref115] Le Ru E.; Etchegoin P. Rigorous justification of the| E| 4 enhancement factor in surface enhanced Raman spectroscopy. Chem. Phys. Lett. 2006, 423, 63–66. 10.1016/j.cplett.2006.03.042.

[ref116] Arenas J. F.; López Tocón I.; Otero J. C.; Marcos J. I. Charge transfer processes in surface-enhanced raman scattering. Franck- condon active vibrations of pyridine. J. Phys. Chem. 1996, 100, 9254–9261. 10.1021/jp953712y.

[ref117] Khaing Oo M. K.; Guo Y.; Reddy K.; Liu J.; Fan X. Ultrasensitive vapor detection with surface-enhanced Raman scattering-active gold nanoparticle immobilized flow-through multihole capillaries. Anal. Chem. 2012, 84, 3376–3381. 10.1021/ac300175v. 22413933

[ref118] Dresselhaus M. S.; Jorio A.; Hofmann M.; Dresselhaus G.; Saito R. Perspectives on carbon nanotubes and graphene Raman spectroscopy. Nano Lett. 2010, 10, 751–758. 10.1021/nl904286r. 20085345

[ref119] Ling X.; Xie L.; Fang Y.; Xu H.; Zhang H.; Kong J.; Dresselhaus M. S.; Zhang J.; Liu Z. Can. graphene be used as a substrate for Raman enhancement?. Nano Lett. 2010, 10, 553–561. 10.1021/nl903414x. 20039694

[ref120] Voloshina E.; Mollenhauer D.; Chiappisi L.; Paulus B. Theoretical study on the adsorption of pyridine derivatives on graphene. Chem. Phys. Lett. 2011, 510, 220–223. 10.1016/j.cplett.2011.05.025.

[ref121] Kim J.; Son H.; Cho D. J.; Geng B.; Regan W.; Shi S.; Kim K.; Zettl A.; Shen Y.-R.; Wang F. Electrical control of optical plasmon resonance with graphene. Nano Lett. 2012, 12, 5598–5602. 10.1021/nl302656d. 23025816

[ref122] Thongrattanasiri S.; Garcia de Abajo F. J. Optical field enhancement by strong plasmon interaction in graphene nanostructures. Phys. Rev. Lett. 2013, 110, 187401 10.1103/PhysRevLett.110.187401. 23683241

[ref123] Van Duin A. C.; Dasgupta S.; Lorant F.; Goddard W. A. ReaxFF: a reactive force field for hydrocarbons. J. Phys. Chem. A 2001, 105, 9396–9409. 10.1021/jp004368u.

[ref124] Chenoweth K.; Van Duin A. C.; Goddard W. A. ReaxFF reactive force field for molecular dynamics simulations of hydrocarbon oxidation. J. Phys. Chem. A 2008, 112, 1040–1053. 10.1021/jp709896w. 18197648

[ref125] SCM. ReaxFF 2021.1; Theoretical Chemistry; Vrije Universiteit: Amsterdam, The Netherlands. http://www.scm.com.

[ref126] Monti S.; Corozzi A.; Fristrup P.; Joshi K. L.; Shin Y. K.; Oelschlaeger P.; Van Duin A. C.; Barone V. Exploring the conformational and reactive dynamics of biomolecules in solution using an extended version of the glycine reactive force field. Phys. Chem. Chem. Phys. 2013, 15, 15062–15077. 10.1039/c3cp51931g. 23925839

[ref127] Golkaram M.; van Duin A. C. Revealing graphene oxide toxicity mechanisms: A reactive molecular dynamics study. Mater. Discovery 2015, 1, 54–62. 10.1016/j.md.2015.10.001.

[ref128] Martyna G. J.; Klein M. L.; Tuckerman M. Nosé–Hoover chains: The canonical ensemble via continuous dynamics. J. Chem. Phys. 1992, 97, 2635–2643. 10.1063/1.463940.

[ref129] Martyna G. J.; Tobias D. J.; Klein M. L. Constant pressure molecular dynamics algorithms. J. Chem. Phys. 1994, 101, 4177–4189. 10.1063/1.467468.

[ref130] Umadevi D.; Sastry G. N. Impact of the chirality and curvature of carbon nanostructures on their interaction with aromatics and amino acids. ChemPhysChem 2013, 14, 2570–2578. 10.1002/cphc.201300089. 23650176

[ref131] Daura X.; Gademann K.; Jaun B.; Seebach D.; Van Gunsteren W. F.; Mark A. E. Peptide folding: when simulation meets experiment. Angew. Chem., Int. Ed. 1999, 38, 236–240. 10.1002/(SICI)1521-3773(19990115)38:1/2<236::AID-ANIE236>3.0.CO;2-M.

[ref132] Abraham M. J.; Murtola T.; Schulz R.; Páll S.; Smith J. C.; Hess B.; Lindahl E. GROMACS: High performance molecular simulations through multi-level parallelism from laptops to supercomputers. SoftwareX 2015, 1, 19–25. 10.1016/j.softx.2015.06.001.

[ref133] Abraham L.; Hess B.; Spoel V. GROMACS 2020.3 source code; Zenodo, 2020.

[ref134] Abdelsalam M. E.; Bartlett P. N.; Baumberg J. J.; Cintra S.; Kelf T. A.; Russell A. E. Electrochemical SERS at a structured gold surface. Electrochem. Commun. 2005, 7, 740–744. 10.1016/j.elecom.2005.04.028.

