# Supplementary material for: QM/Classical Modeling of Surface Enhanced Raman Scattering Based on Atomistic Electromagnetic Models
Source: J Chem Theory Comput. 2023 Jun 6;19(12):3616–33. doi: 10.1021/acs.jctc.3c00177 (PMC10308587; doi:10.1021/acs.jctc.3c00177)
Supplement: Supplementary file 1 — ct3c00177_si_001.pdf [file ct3c00177_si_001.pdf]

Supporting Information: QM/Classical Modeling of Surface Enhanced Raman Scattering Based on Atomistic Electromagnetic Models

Piero Lafiosca,[†] Luca Nicoli,[†] Luca Bonatti,[†] Tommaso Giovannini,[*,†] Stefano Corni,[‡,¶] and Chiara Cappelli[*,†,§]

†*Scuola Normale Superiore, Piazza dei Cavalieri 7, 56126 Pisa, Italy.*

‡*Dipartimento di Scienze Chimiche, Università di Padova, via Marzolo 1, 35131, Padova, Italy*

¶*Istituto di Nanoscienze del Consiglio Nazionale delle Ricerche CNR-NANO, via Campi 213/A, 41125, Modena, Italy*

§*LENS (European Laboratory for Non-Linear Spectroscopy), Via N. Carrara 1, 50019, Sesto Fiorentino, Italy*

E-mail: tommaso.giovannini@sns.it; chiara.cappelli@sns.it

Table S1: List of abbreviations used in the manuscript

ωFQ	Frequency dependent Fluctuating Charges
ωFQFμ	Frequency dependent Fluctuating Charges and Fluctuating Dipoles
EM	electromagnetic
CT	chemical enhancement
NP	metal nanoparticles
IB	interband (transitions)
PS	plasmonic substrate
cTO	cuboctahedron
Ih	icosahedron
i-Dh	ino-decahedron
GD	graphene disk
PY	pyridine
PRF	plasmon resonance frequency
EF	enhancement factor
AEF	(spectrally) averaged enhancement factor
MEF	maximum enhancement factor
MTX	methotrexate
PY-V	pyridine adsorbed on the vertex of a plasmonic substrate
PY-E	pyridine adsorbed on the edge of a plasmonic substrate
PY-F	pyridine adsorbed on the face of a plasmonic substrate
MTX1, MTX2	conformers of methotrexate

Table S2: Geometrical parameters (number of atoms and radius) of Ag and Au nanoparticles used in this work. The associated plasmon resonance frequencies (PRF) are also given.

Shape	Number of atoms	Radius (Å)	PRF (eV)	
			Ag	Au
cTO	147	8.66	3.54	-
	309	11.54	3.49	-
	561	14.43	3.46	2.27
	923	17.31	3.47	-
	1415	20.20	3.45	2.23
	2057	23.08	3.44	2.21
	2869	25.97	3.43	2.20
	3871	28.85	3.43	2.19
	5083	31.74	3.43	2.17
	6525	34.62	3.43	2.17
	8217	37.51	3.42	2.17
	10179	40.39	3.42	2.17

Ih	147	8.23	3.64	-
	309	10.98	3.58	-
	561	13.72	3.56	-
	923	16.46	3.54	-
	1415	19.21	3.53	2.29
	2057	21.95	3.52	2.26
	2869	24.69	3.52	2.24
	3871	27.44	3.51	2.23
	5083	30.13	3.51	2.21
	6525	32.87	3.51	2.21
	8217	35.57	3.51	2.21
	10179	38.30	3.51	2.21
i-Dh	85	5.770	3.52	-
	207	8.655	3.48	-
	409	11.54	3.47	-
	711	14.43	3.46	2.31
	1133	17.31	3.45	2.25
	1695	20.20	3.44	2.22
	2417	23.08	3.44	2.20
	3319	25.97	3.43	2.19
	4421	28.85	3.43	2.18
	5743	31.74	3.43	2.18
	7305	34.62	3.42	2.18
	9127	37.50	3.42	2.17

Table S3: Geometrical parameters (number of atoms and radius) of graphene disks used in this work. The associated plasmon resonance frequencies (PRF) are also given.

Shape	Label	Number of atoms	Radius (Å)	PRF (eV)
GD	GD2	469	20	0.61
	GD4	1909	40	0.46
	GD6	4294	60	0.38
	GD8	7669	80	0.33
	GD10	11980	100	0.30
	GD12	17269	120	0.28
	GD14	23485	140	0.26
	GD16	30724	160	0.24

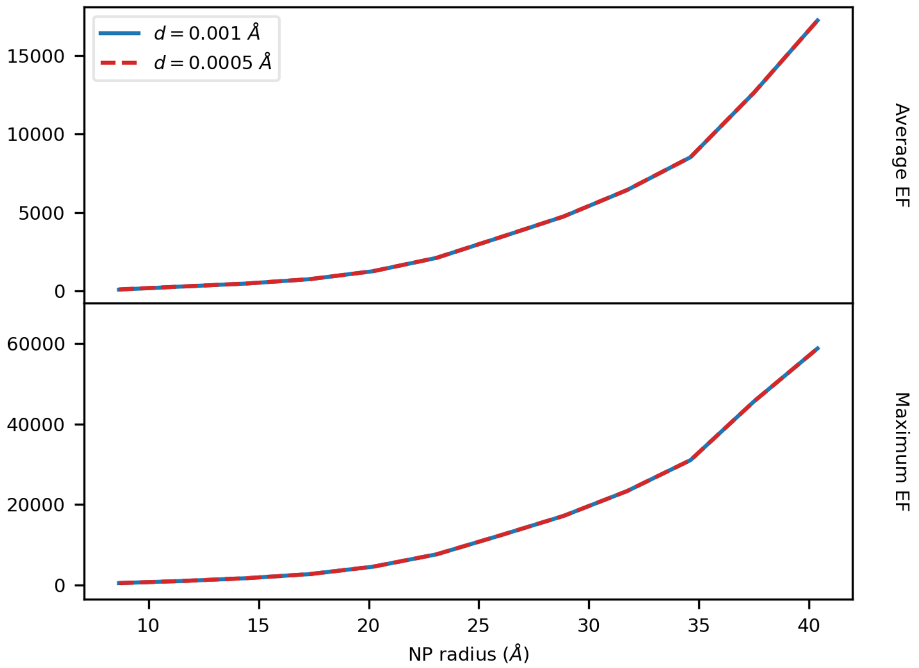

Figure S1: Dependence of AEF and MEF on the numerical differentiation step (d=0.001 Å or d=0.0005 Å) used in the calculation of complex polarizability derivatives. PY adsorbed on Ag cTO systems.

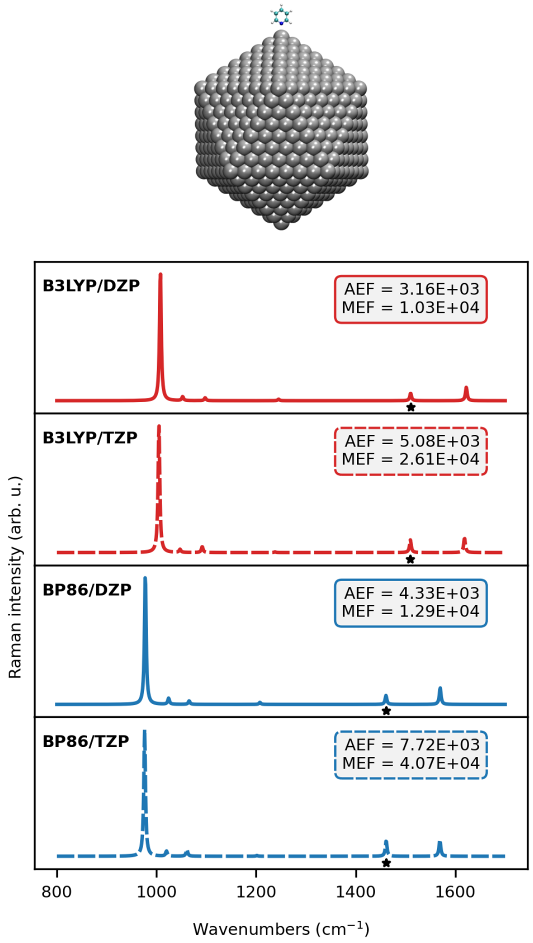

(a) Silver

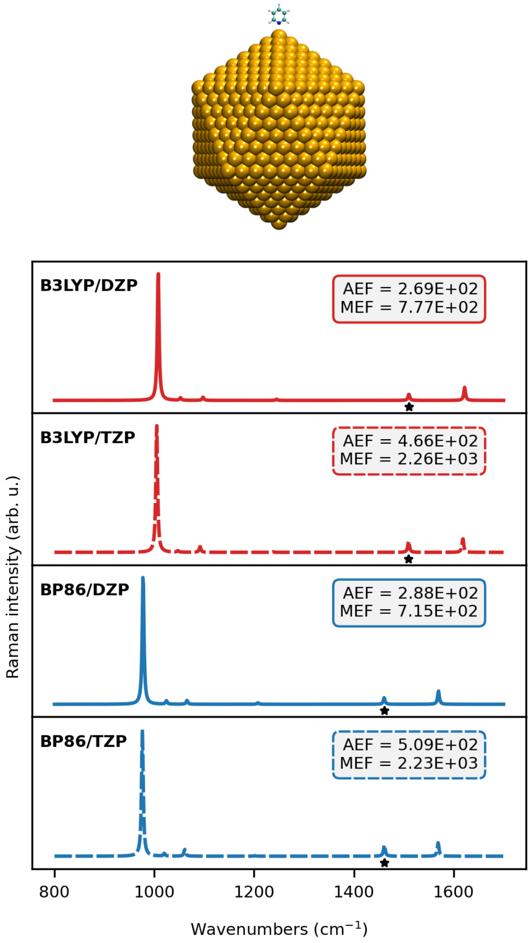

(b) Gold

Figure S2: Dependence of SERS spectra of PY adsorbed on Ih Ag_{10179} (left) and Au_{10179} (left) on the level of theory. The vibrational frequency associated with the MEF is highlighted by a black star.

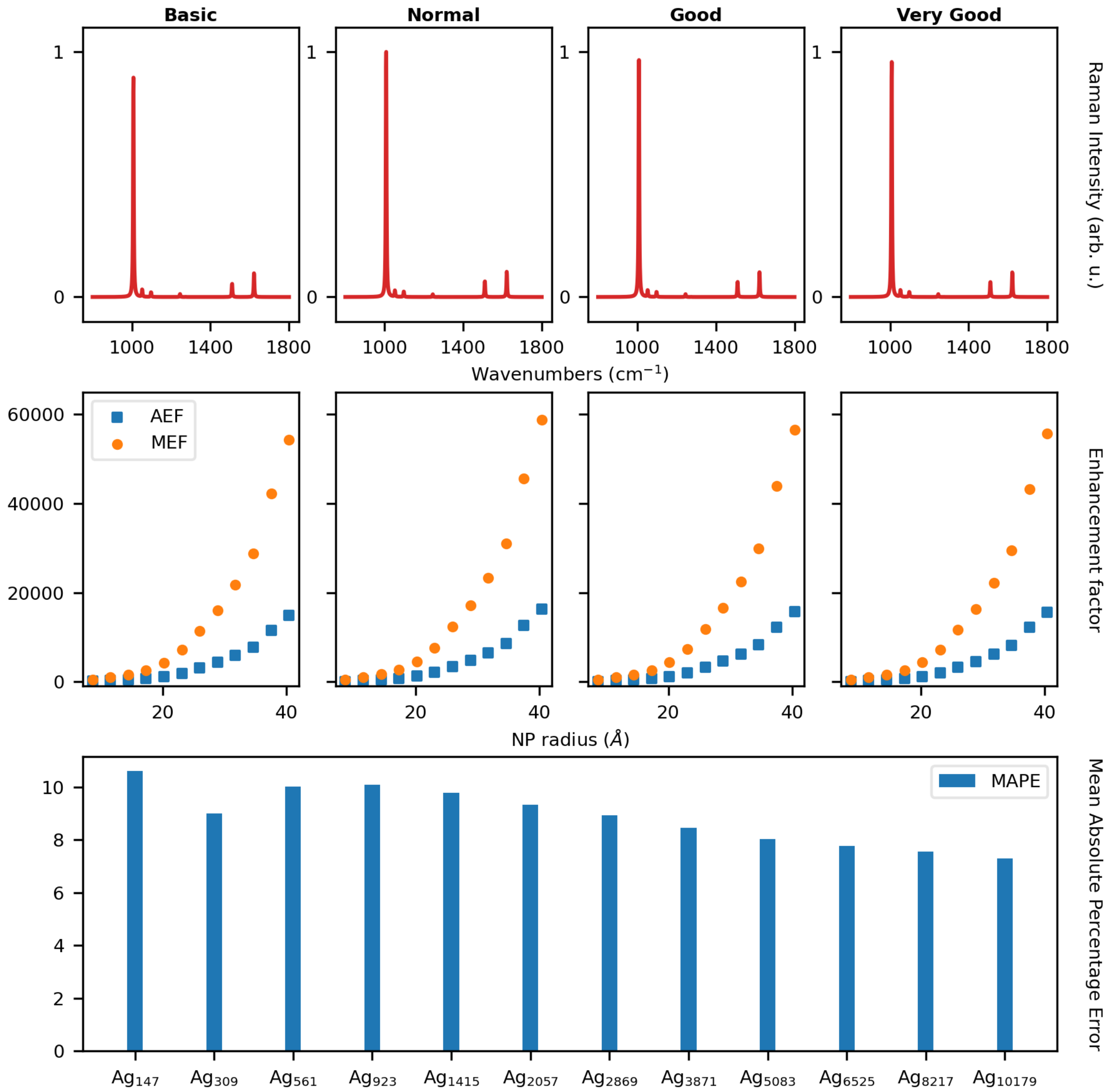

Figure S3: QM/ωFQFμ calculations on the pyridine/Ag system in the cTO morphology by exploiting DFT integration grids of increasing accuracy. Top panel: normalized SERS spectra of pyridine adsorbed on the largest cTO structure (Ag_{10179}). Middle panel: AEF and MEF as a function of the NP radius. Bottom panel: Mean Absolute Percentage Error (MAPE) of EFs, when the latter is computed with "Numerical Quality Normal" with respect to "Numerical Quality VeryGood" for NPs of various size. All calculations are performed at the B3LYP/DZP level of theory.

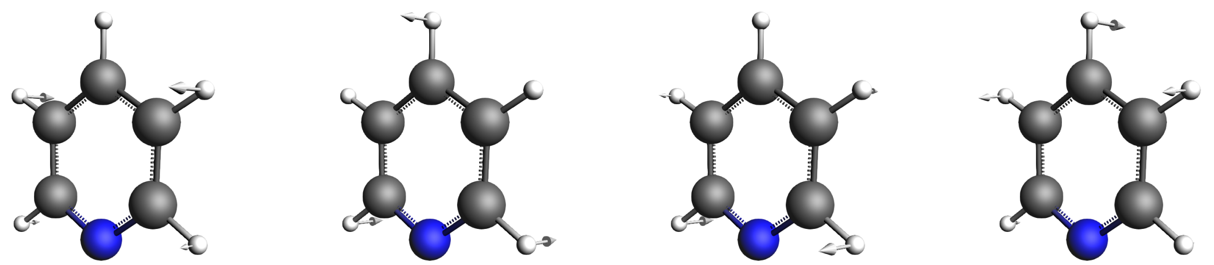

(a) 899 cm-1 (b) 956 cm-1 (c) 1000 cm-1 (d) 1008 cm-1

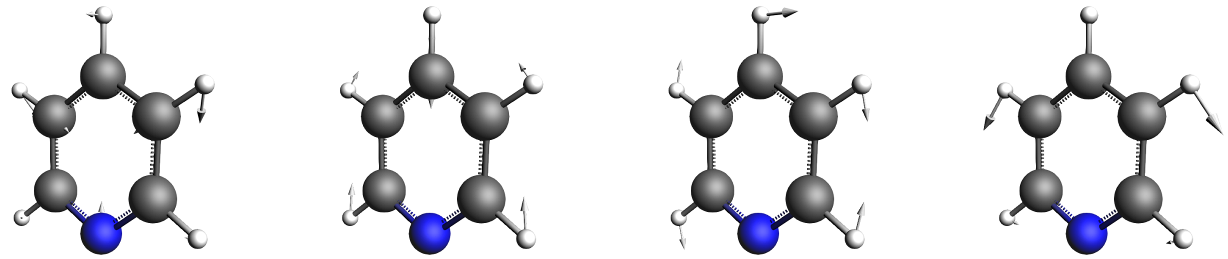

(e) 1008 cm-1 (f) 1053 cm-1 (g) 1078 cm-1 (h) 1098 cm-1

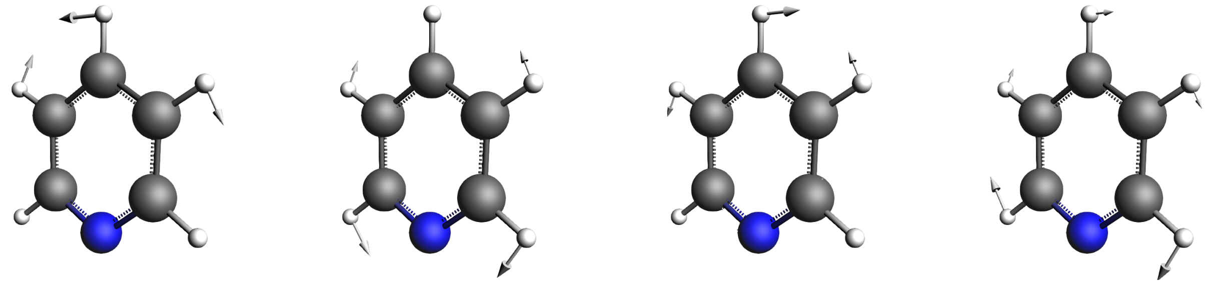

(i) 1181 cm-1 (j) 1245 cm-1 (k) 1274 cm-1 (l) 1388 cm-1

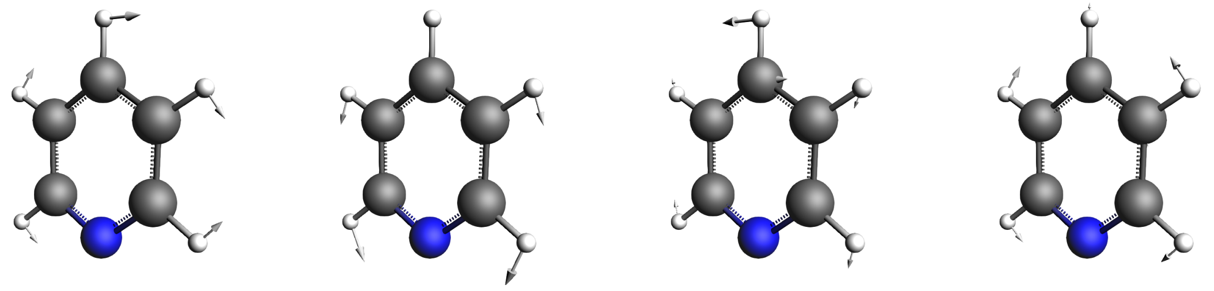

(m) 1473 cm-1 (n) 1510 cm-1 (o) 1616 cm-1 (p) 1622 cm-1

Figure S4: Vibrational normal modes of PY in vacuo calculated at B3LYP/DZP level of theory.

Table S4: Enhancement factors of PY adsorbed on Ag_{10179}, Au_{10179} and GD4.

Wavenumber (cm^{-1})	Ag			Au			GD
	cTO	Ih	i-Dh	cTO	Ih	i-Dh	$R = 40$ Å
898.64	1.35	0.94	2.17	0.04	0.04	0.04	2244.46
956.44	197.71	148.12	226.37	14.18	9.42	8.13	70093.32
999.92	0.90	0.78	1.55	0.01	0.00	0.01	871.76
1007.84	34527.07	6705.84	8218.10	666.05	568.87	443.33	14329.97
1007.85	34828.44	6769.42	8297.90	769.05	573.70	512.51	10043.62
1052.58	1098.16	258.18	427.98	18.90	14.33	15.56	10947.63
1078.03	416.86	57.03	114.94	2.26	1.01	1.47	11209.79
1097.74	30026.28	5966.64	7533.43	714.95	539.00	485.58	8956.54
1181.17	136.27	34.49	53.14	3.44	3.14	2.84	10502.80
1245.18	1718.09	370.70	573.17	33.54	23.59	24.20	9723.70
1274.20	201.59	32.92	55.93	2.70	1.93	1.99	9395.24
1387.78	1993.79	333.95	530.69	0.98	1.21	0.63	12150.37
1472.56	146.16	45.13	43.48	4.47	3.99	3.37	12347.82
1510.30	58738.36	10286.44	11988.96	1106.07	776.91	671.46	10587.33
1615.76	253.79	60.30	93.51	4.11	3.85	3.41	11134.33
1622.08	15235.88	2980.92	3850.27	360.65	268.83	242.29	11183.74
AEF	15218.65	2954.27	3681.90	334.26	249.00	222.84	10520.91

Table S5: Average Enhancement Factors (AEF) and absolute Υ^4_{vol} for Ag nanostructures. The external field is directed along the pyridine molecular axis.

Structure	cTO		Ih		i-Dh	
	AEF	Υ^4_{vol}	AEF	Υ^4_{vol}	AEF	Υ^4_{vol}
1	71.49	20842.90	33.48	11764.20	10.41	11764.20
2	270.54	32820.81	136.26	20015.08	43.61	20015.08
3	458.77	55400.45	28.86	32425.23	136.97	32425.23
4	737.30	91651.60	135.04	50134.76	269.37	50134.76
5	1246.36	147054.50	377.41	76897.12	423.28	76897.12
6	2098.16	228347.38	567.14	114971.62	628.98	114971.62
7	3407.28	338220.33	733.55	170596.26	862.55	170596.26
8	4738.77	509599.52	1279.95	242233.09	1261.68	242233.09
9	6427.08	737693.47	1643.09	341402.32	1682.09	155968.96
10	8525.95	1032631.53	2074.32	466134.52	2203.65	222169.56
11	12646.52	1357815.07	2579.17	619243.56	3085.70	619243.56
12	16269.71	1819201.17	3163.13	803358.62	3936.84	803358.62

Table S6: Average Enhancement Factors (AEF) and absolute Υ^4_{vol} for graphene nanostructures. The external field is directed along the graphene plane.

Structure	AEF	Υ^4_{vol}
1	9375.43	11260.83
2	10402.45	11876.37
3	6687.45	8079.50
4	4115.87	5417.91
5	3278.32	3696.09
6	2550.34	2153.59
7	1976.16	1666.88
8	1509.64	1605.28

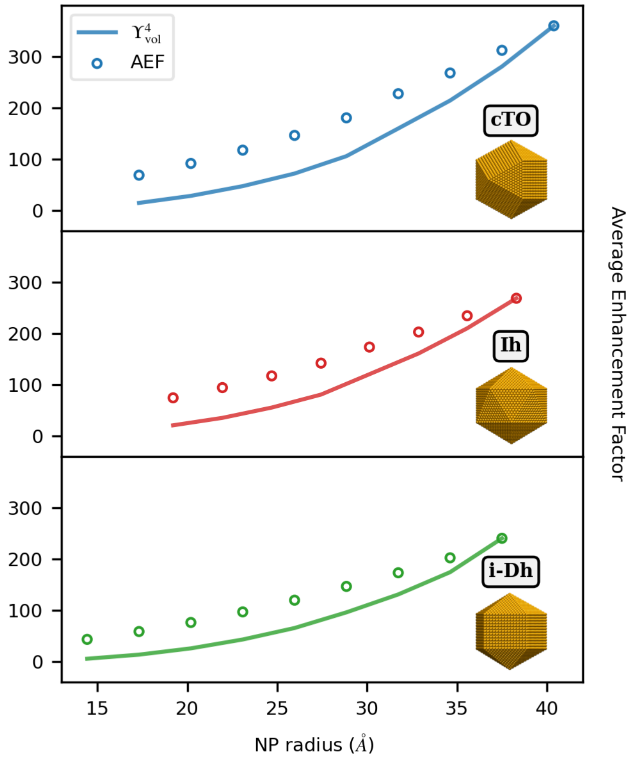

Figure S5: AEF (circles) and Υ^4_{vol} (solid line) as a function of the Au NP radius.

Table S7: Average Enhancement Factors (AEF) and absolute $\Upsilon^4_{\mathrm{vol}}$ for different values of the PY N-Ag_{10179} distance. The external field is directed along the pyridine molecular axis.

Distance (Å)	AEF	$\Upsilon^4_{\mathrm{vol}}$
3.0	3163.13	803358.62
3.5	1278.98	576391.98
4.0	715.43	419288.14
4.5	448.03	309808.78
5.0	302.43	232696.42
5.5	214.56	177537.96
6.0	157.86	137394.02
7.0	92.49	85348.18
8.0	58.66	55199.50
9.0	39.45	36888.75
10.0	27.81	25331.77

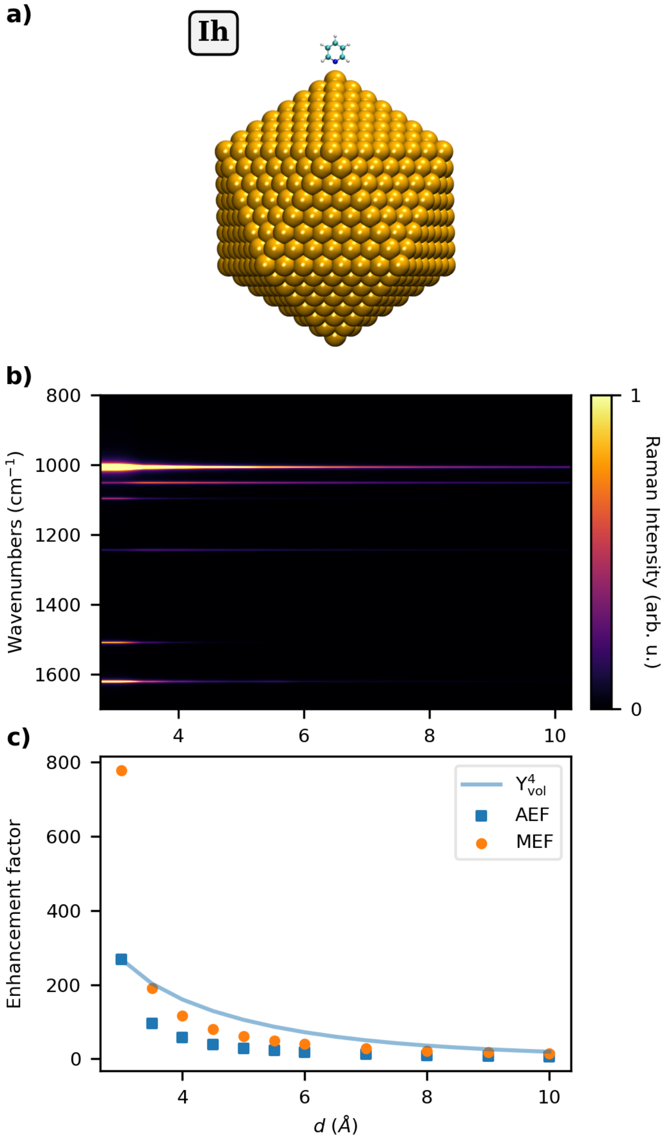

Figure S6: a) Graphical depiction of PY-Au_{10179} Ih system; (b) color plot of normalized SERS spectra as a function of the PY-NP distance d (Å); (c) AEF, MEF and normalized Υ^4_{vol} as a function of d.

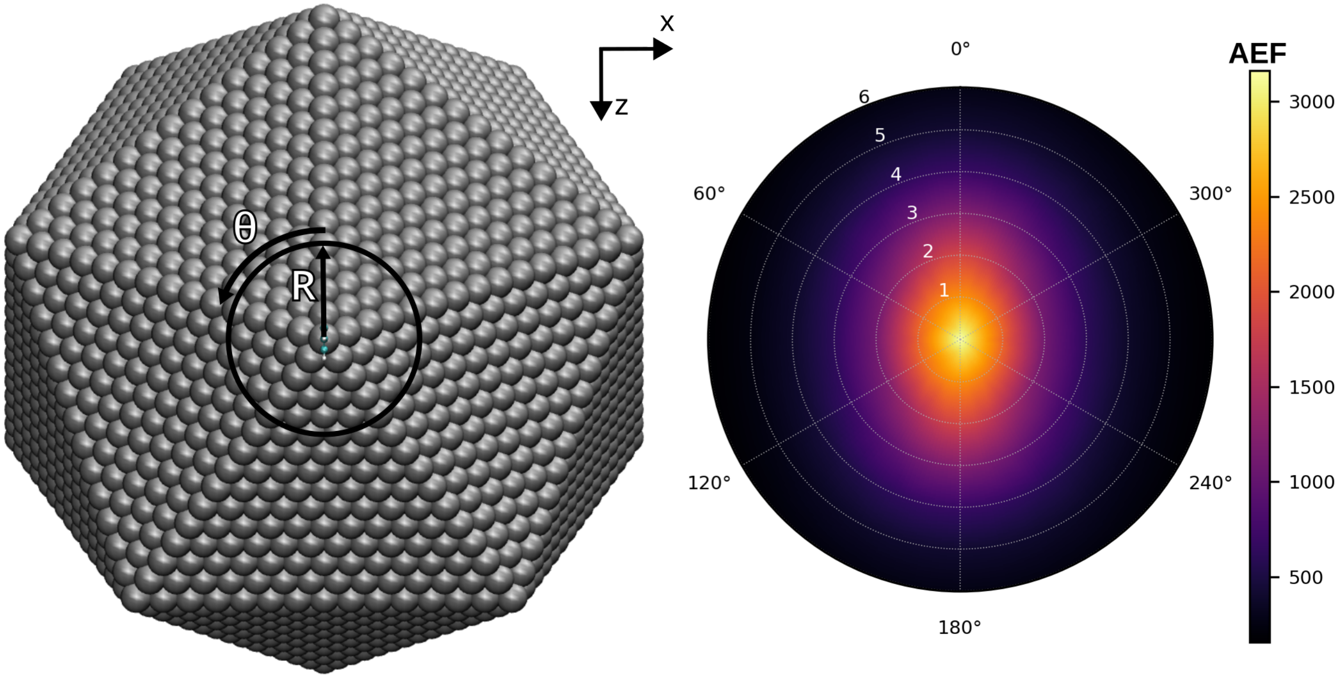

Figure S7: Dependence of the SERS signal on the PY-Ag_{10179} position (Ih morphology). Left panel: graphical depiction of the PY-Ag system with indication of the polar coordinates system. Right panel: AEF of the PY-Ag system computed at different values of R (from 1 to 6 Å with a step of 1 Å) and θ (from 0 to 180 degrees with a step of 10 degrees). The values for $\theta = 180$ degrees to $\theta = 360$ degrees have been obtained by reflection with respect to the yz plane, for symmetry reasons.

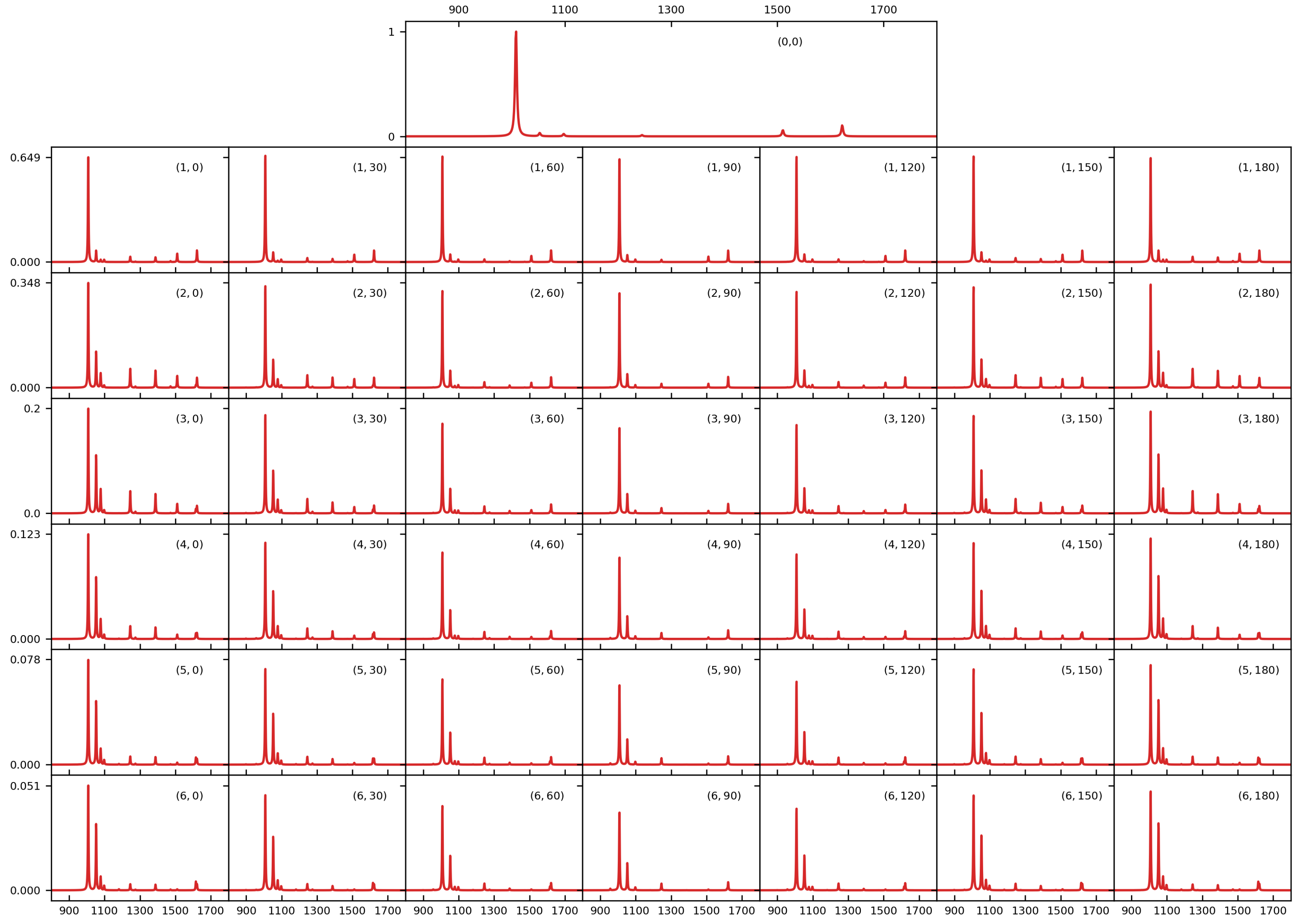

Figure S8: SERS spectra computed at different PY-Ag_{10179} positions (Ih morphology). Each spectrum is obtained at the specified values of (R, θ) coordinates (see Fig. S7 for the definition). The reference spectrum (Py on the tip of the Ih NP) is reported in the first row ($(R, \theta) = (0, 0)$).

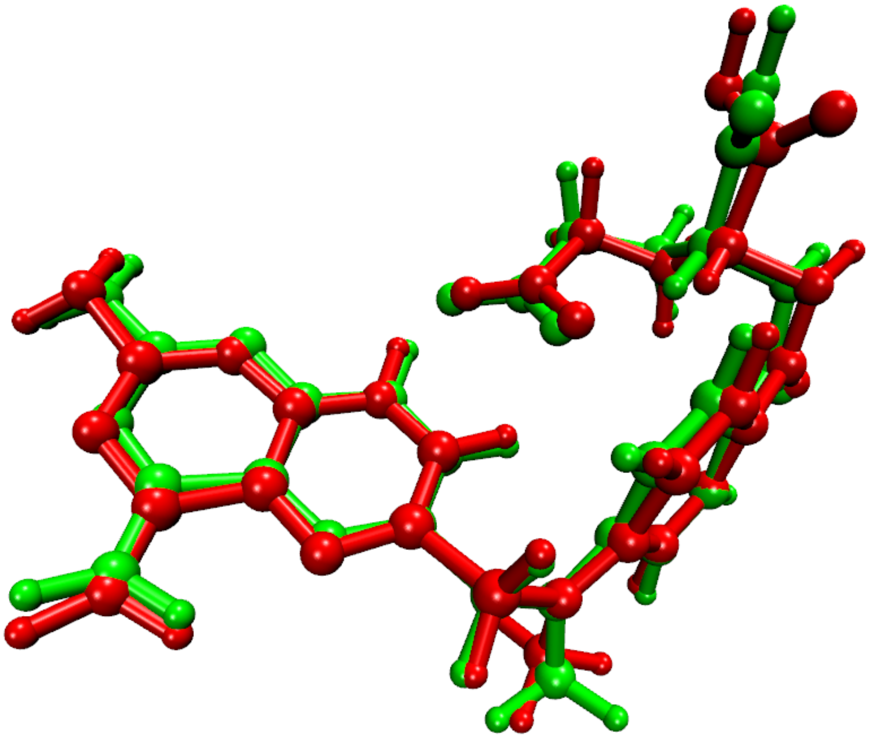

Figure S9: Superposition of MTX1 and MTX2 geometries (see main text).

S1 Dependence of AEF on electric field intensity

Let us consider a molecular system adsorbed on a plasmonic substrate, both interacting with an external field aligned along the z direction. The Raman signal of the molecular vibrational normal modes is enhanced by the presence of the plasmonic substrate as a result of the increase of the incident and/or scattered fields. The vibrational normal modes can be partitioned into three subsets:

- The vibrational normal modes Q_a (total number N_a) whose intensity is dominated by the contribution $\left|\frac{\partial \overline{\alpha}_{zz}(\omega;\omega)}{\partial Q_a}\right|^2$, which are enhanced by the plasmonic substrate via both the incident and scattered fields, yielding a total enhancement factor of $|E|^4$;
- The vibrational normal modes Q_b (total number N_b) whose intensity is dominated by the contribution $\left|\frac{\partial \overline{\alpha}_{iz}(\omega;\omega)}{\partial Q_b}\right|^2$ or $\left|\frac{\partial \overline{\alpha}_{zi}(\omega;\omega)}{\partial Q_b}\right|^2$ with $i \neq z$, which are enhanced by the

plasmonic substrate via the incident or the scattered fields only, yielding a total enhancement factor of $|E|^2$;

- The vibrational normal modes Q_c (total number N_c) whose intensity is dominated by the contribution $\left|\frac{\partial\overline{\alpha}_{ij}(\omega;\omega)}{\partial Q_c}\right|^2$ with $i, j \neq z$, which are not enhanced by the plasmonic substrate.

Therefore, the AEF can be computed as follows:

$$\mathrm{AEF} = \frac{N_a|E|^4 + N_b|E|^2 + N_c}{N_a + N_b + N_c} \overset{|E|^2 \gg 1}{\approx} |E|^4 \frac{N_a}{N_a + N_b + N_c},$$

where the second equality is true when $|E|^2 \gg 1$, thus $|E|^4 \gg |E|^2$. Therefore, the AEF follows the E^4 approximation for strong electric field intensities.
